# Human monocyte-derived macrophages shift subcellular metalloprotease activity depending on their activation state

**DOI:** 10.1016/j.isci.2024.111171

**Published:** 2024-10-16

**Authors:** Eline Bernaerts, Kourosh Ahmadzadeh, Amber De Visscher, Bert Malengier-Devlies, Daniel Häuβler, Tania Mitera, Erik Martens, Achim Krüger, Lien De Somer, Patrick Matthys, Jennifer Vandooren

**Affiliations:** 1 Laboratory of Immunobiology, Rega Institute for Medical Research, KU Leuven, 3000 Leuven, Belgium; 2 Centre for Reproductive Health and Centre for Inflammation Research, Institute for Regeneration and Repair, University of Edinburgh, Edinburgh EH16 4UU, UK; 3 TUM School of Medicine and Health, Institute of Experimental Oncology and Therapy Research, Technical University of Munich, D-81676 Munich, Germany; 4 University Hospital Leuven, Laboratory of Pediatric Immunology, 3000 Leuven, Belgium; 5 Department of Microbiology, Immunology and Transplantation, KU Leuven Campus Kulak, 8500 Kortrijk, Belgium

**Keywords:** Natural sciences, Biological sciences, Immunology, Immune response

## Abstract

Proteases are key effectors in macrophage function during the initiation and resolution of inflammation. Recent studies have shown that some proteases, traditionally considered extracellular, also exhibit enzymatic and non-enzymatic functions within the cell. This study explores the differential protease landscapes of macrophages based on their phenotype. Human monocytes were isolated from healthy volunteers and stimulated with M-CSF (resting macrophages), LPS/IFN-γ (inflammatory macrophages), or IL-4 (immunosuppressive macrophages). IL-4-stimulated macrophages secreted higher levels of MMPs and natural protease inhibitors compared to LPS/IFN-γ-stimulated macrophages. Increased extracellular proteolytic activity was detected in LPS/IFN-γ-stimulated macrophages while IL-4 stimulation increased cell-associated proteolytic activity, particularly for MMPs. Subcellular fractionation and confocal microscopy revealed the uptake of extracellular MMP-9 and its relocation to the nucleus in IL-4-stimulated, though not in LPS/IFN-γ-stimulated macrophages. Collectively, macrophages alter the subcellular location and activity of their MMPs based on the stimuli received, suggesting another mechanism for protease regulation in macrophage biology.

## Highlights

• Macrophages alter MMP location and proteolytic activity based on received stimuli
• LPS/IFN-γ stimulation increases extracellular proteolytic activity
• IL-4 stimulation heightens macrophage-associated proteolytic activity
• MMP-9 relocates to the nucleus upon IL-4 stimulation, unlike LPS/IFN-γ stimulation

## Graphical abstract

**Figure:**
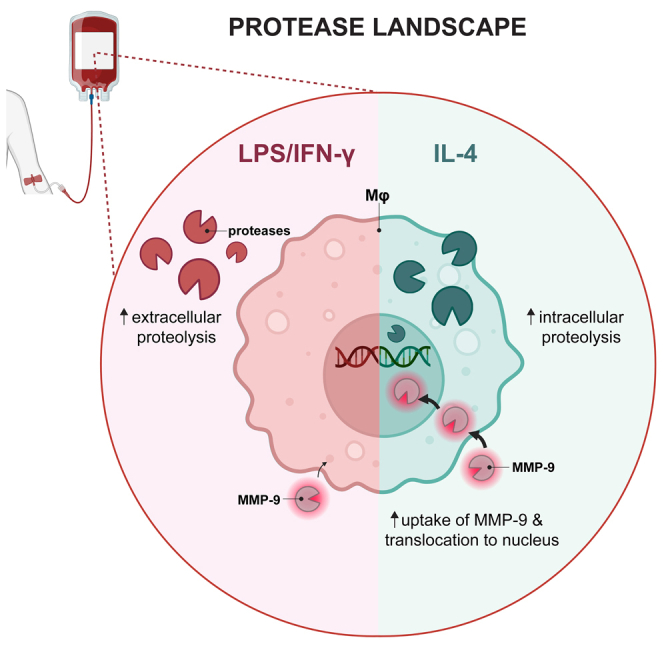

## Introduction

Macrophages play an important role in the initiation and resolution of inflammation. Due to their plasticity, macrophages can differentiate into distinct phenotypes, ranging from inflammatory to immunosuppressive, depending on their exposure to local micro-environmental stimuli.^1^^,^^2^ Upon stimulation with interferon gamma (IFN-γ), primarily secreted by activated T cells and natural killer (NK) cells, and with lipopolysaccharide (LPS) derived from bacteria, macrophages typically polarize into an inflammatory response phenotype. This process results in the increased production of reactive oxygen species (ROS) and pro-inflammatory cytokines (e.g., IL-1β, IL-6).^3^^,^^4^ In contrast, the stimulation of macrophages with IL-4 promotes their production of anti-inflammatory mediators (e.g., IL-10), giving rise to immunosuppressive macrophages.^1^^,^^5^^,^^6^^,^^7^ However, the exact mechanisms driving macrophage phenotypic changes and the composition and importance of different macrophage phenotypes in inflammation are not yet fully understood.

Proteolysis is a key process in the distinct effector functions of macrophages.^2^^,^^8^^,^^9^ The migration and invasion of monocytes and macrophages to the site of inflammation are assisted by the proteases they produce.^10^ 2D/3D migration, diapedesis, and migration across blood-brain barriers require the action of matrix metalloproteinases (MMPs), cathepsins, and urokinase-type plasminogen activator (uPA) through the remodeling of the extracellular matrix.^9^^,^^11^ Once on-site, they help clear and digest tissue material^12^ and modulate the immune response via the cleavage of cytokines, chemokines, and their cognate receptors.^13^^,^^14^ The secretion of proteases is accompanied by the secretion of natural protease inhibitors such as tissue inhibitors of metalloproteinases (TIMP)-1 and TIMP-2, cystatins and serpins, as a means to balance proteolytic activity in the microenvironment and the resulting biological activities.^15^ Proteases can furthermore activate or inactivate the expression of other proteases or cleave inhibitors thereby modulating their activity and contributing to an intricate proteolytic network.^16^^,^^17^ Investigating the network of proteases, their regulatory molecules (e.g., inhibitors) and the resulting net proteolytic activity can be challenging, but this is a prerequisite to gain a better understanding of the proteolytic environment. Furthermore, recent studies suggest that the subcellular location of proteases contributes to their biological functioning. In fact, extracellular proteases have been identified within cells, exerting enzymatic activities such as the cleavage of transcription factors, and distinct non-enzymatic functions such as signal transduction and regulating gene expression.^18^^,^^19^ In macrophages, nuclear MMP-12 can inhibit the expression of different genes, such as those encoding for proteasome activator complex subunit 3 and SPARC-like protein-1, whereas extracellular MMP-12 can attenuate the immune response by cleaving IFN-α.^20^ As another example, nuclear MMP-14 can regulate the inflammatory immune response, by activating the transcription of phosphoinositide-3 kinase-ζ. This allows macrophages to transition from a pro-inflammatory to an anti-inflammatory immune response.^21^ Hence, the relocation of macrophage proteases holds promise as a strategy to regulate macrophage functions.

Reports on the systematic investigation of proteases and their regulators in different activation states of human macrophages and their implications in inflammation are scarce. Some studies have revealed differences in mRNA expression levels of proteases and their regulators in mouse bone marrow-derived macrophages and macrophages derived from cell lines.^22^^,^^23^^,^^24^^,^^25^^,^^26^^,^^27^ However, research on protein levels, subcellular location and corresponding net activity in human primary macrophages, depending on the stimuli received, has not been thoroughly investigated. Additionally, studying macrophage proteases in the absence of exogenous proteases and inhibitors (derived from culture medium containing FBS) has been challenging and is often overlooked. To explore the potential role of proteases secreted by inflammatory and immunosuppressive macrophages, which are critical during the initiation and resolution of inflammation, we studied the evolution of the protease landscape of macrophages in relation to commonly studied phenotypes, in the absence of exogenous proteases and inhibitors.

## Results

### Generation of activated macrophages in the absence of exogenous proteases and inhibitors

**Figure 1:**
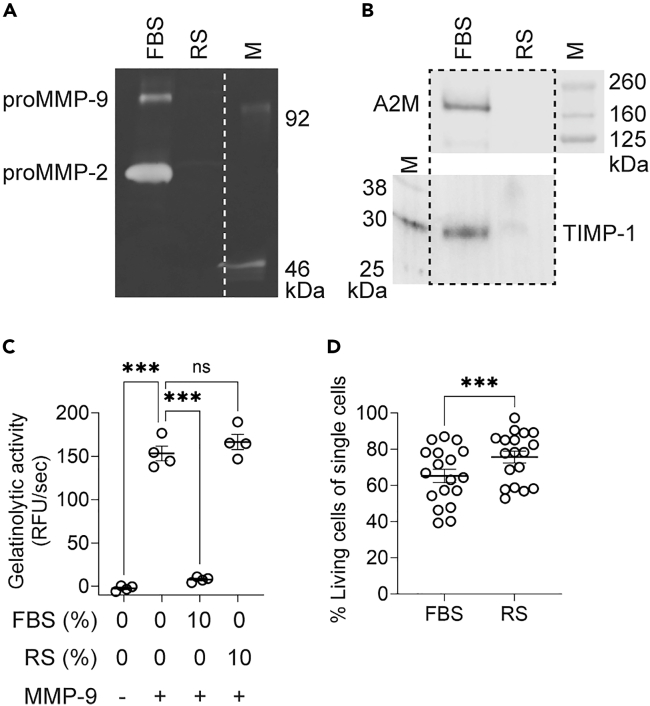
Generation of activated macrophages in the absence of exogenous proteases and inhibitors (A) Gelatin *in gel* zymography analysis of cell culture medium with FBS or replacement serum (RS). Marker (M) indicates monomeric proMMP-9 and a low-molecular weight proMMP-9 domain deletion mutant lacking the O-glycosylated and hemopexin domains (proMMP-9 ΔOGΔHem). (B) Western blot analysis of A2M and TIMP-1 in cell culture medium with FBS or RS. (C) Gelatinolytic activity of MMP-9 in the presence of 10% FBS or 10% RS. Each data point represents cells from a different donor (*n* = 4). Error bars are mean ± SEM. *p* values less than 0.05 were considered significant (∗∗∗*p* < 0.001; ns non-significant). (D) Percentage living macrophages when cultured with 10% FBS or 20% RS. Each data point represents cells from a different donor (*n* = 18). Error bars are mean ± SEM. *p* values less than 0.05 were considered significant (∗∗∗*p* < 0.001). See also Figure S1.

We generated three commonly studied macrophage activation states: M(M-CSF) (resting macrophages), M(LPS/IFN-γ) (inflammatory macrophages), and M(IL-4) (immunosuppressive macrophages), using macrophages isolated from human donor-derived peripheral blood mononuclear cells (PBMCs). Culture and activation of human primary macrophages are typically done in a complete medium that contains approximately 10% of FBS. However, FBS itself contains considerable levels of bovine proteases (e.g., proMMP-2 and proMMP-9, Figure 1A) and protease inhibitors (e.g., TIMP-1 and A2M, Figure 1B). Furthermore, the presence of these proteins interfered with the detection of protease activity, as demonstrated by performing an activity assay for MMP-9 in the presence of 10% FBS (Figure 1C).

**Figure 2:**
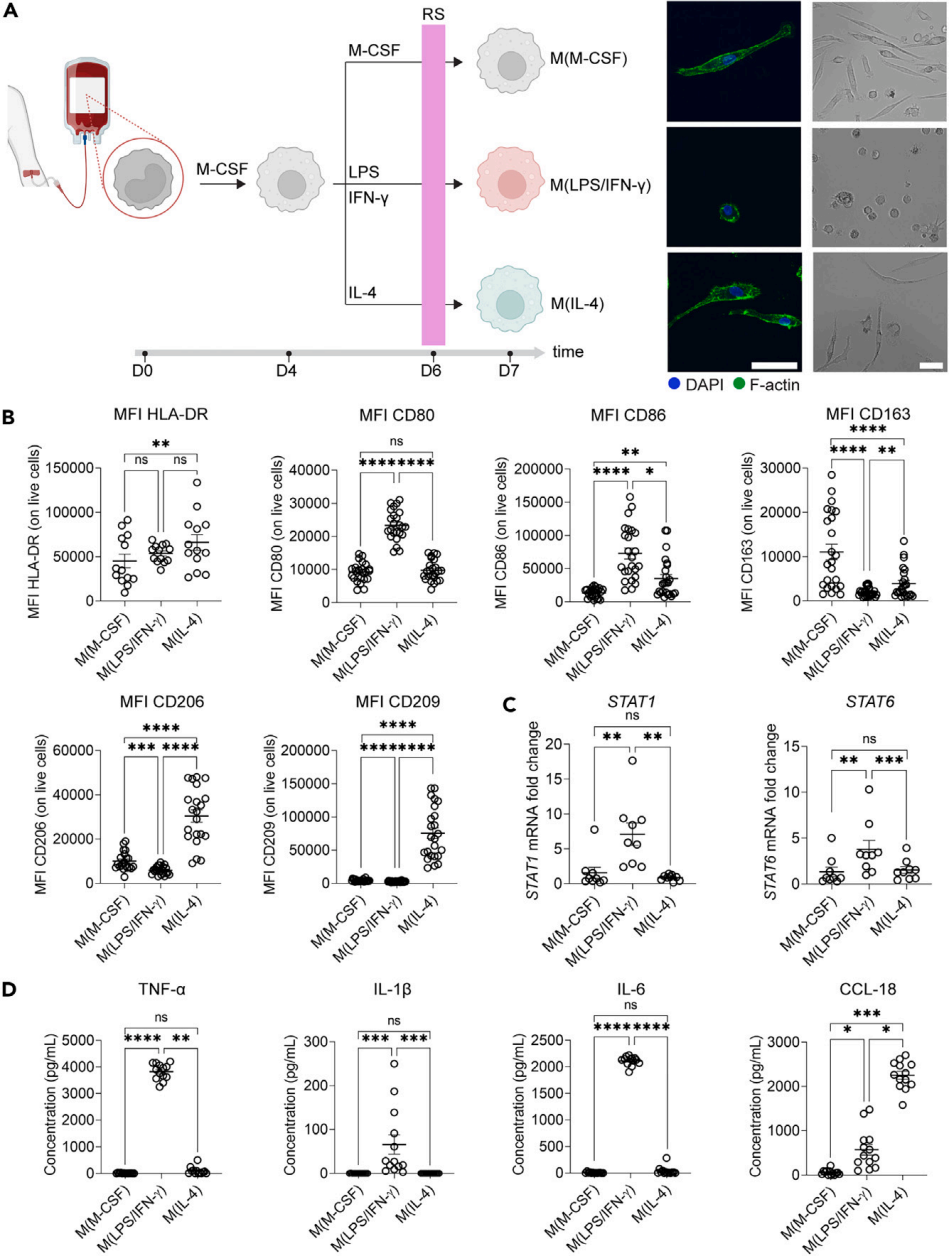
Macrophage phenotype is not altered in cell culture medium lacking FBS (A) Schematic representation of the maturation and stimulation of macrophages. Monocytes from healthy volunteers were first incubated for 4 days with M-CSF/CSF-1 (100 ng/mL). M(M-CSF) were generated by stimulating the macrophages for 3 additional days with M-CSF (25 ng/mL). M(LPS/IFN-γ) were induced by stimulating macrophages for 3 additional days with LPS (50 ng/mL) and IFN-γ (25 ng/mL). M(IL-4) were obtained by stimulating macrophages for 3 additional days with IL-4 (25 ng/mL). Twenty-four hours before macrophage collection and analysis, the differentiation medium was replaced by a medium containing 20% replacement serum (RS). Fluorescence microscopy and light microscopy images of M(M-CSF), M(LPS/IFN-γ), and M(IL-4). Cells were stained for their nuclei (DAPI, blue) and F-actin filaments (Phalloidin, green). Fluorescent images are representative of 4 donors. Light microscopy images are representative of 6 donors. Scale bar represents 100 μm. (B) Flow cytometry analysis of HLA-DR, CD80, CD86, CD163, CD206 and CD209 on M(M-CSF), M(LPS/IFN-γ) and M(IL-4). Each data point represents cells from a different donor (at least *n* = 13). Error bars are mean ± SEM. *p* values less than 0.05 were considered significant (∗*p* < 0.05; ∗∗*p* < 0.01; ∗∗∗*p* < 0.001; ∗∗∗∗*p* < 0.0001; ns non-significant). (C) Relative mRNA expression levels of *STAT1* and *STAT6* of M(M-CSF), M(LPS/IFN-γ), and M(IL-4) as analyzed by qRT-PCR. Each data point represents cells from a different donor (*n* = 9). Error bars are mean ± SEM. *p* values less than 0.05 were considered significant (∗∗*p* < 0.01; ∗∗∗*p* < 0.001; ns non-significant). (D) Quantification of secreted TNF-α, IL-1β, IL-6, and CCL-18 protein levels in cell culture supernatants of M(M-CSF), M(LPS/IFN-γ) and M(IL-4) by ELISA. Each data point represents cells from a different donor (at least *n* = 11). Error bars are mean ± SEM. *p* values less than 0.05 were considered significant (∗*p* < 0.05; ∗∗*p* < 0.01; ∗∗∗*p* < 0.001; ∗∗∗∗*p* < 0.0001; ns non-significant). See also Figure S1.

To prevent interference of protease-containing FBS with our protease assays, we optimized cell culture conditions in the absence of FBS. While 10% FBS was used during the first six days of macrophage differentiation, we changed the cell culture medium 24 h before macrophage analysis and replaced it with an FBS-free growth medium containing 20% replacement serum (Figure 2A), which did not negatively affect cell viability (Figure 1D). Replacement serum is a defined serum-free alternative for cell culture, originally introduced for the culture of embryonic stem cells since factors present in FBS drive their spontaneous differentiation.^28^ In contrast to FBS, no proteases or protease inhibitors could be detected in replacement serum, and it did not interfere with the detection of gelatinolytic activity (Figures 1A–1C). Given that we introduced these changes to the macrophage stimulation protocol, we first performed a phenotypic screen of the obtained macrophages. Fluorescence and light microscopy revealed morphological differences that are typical for inflammatory and immunosuppressive macrophage activation states.^4^^,^^7^^,^^29^^,^^30^ M(M-CSF) showed an elongated morphology. M(LPS/IFN-γ) were smaller cells and showed a round morphology. M(IL-4) was the largest of the three cell types and showed an elongated, expanded morphology with filamentous F-actin staining (Figure 2A). Next, we probed for typical macrophage cell surface markers by flow cytometry (gating strategy in Figure S1). Cell viability of M(M-CSF), M(LPS/IFN-γ), and M(IL-4) were measured by live/dead staining, and no significant differences in cell viability were observed between the three macrophage activation states (Figure S1). Both M(LPS/IFN-γ) and M(IL-4) showed an increased trend of cell-surface HLA-DR expression compared to resting M(M-CSF). However, co-stimulatory ligands for T cell activation, namely CD80 and CD86, were significantly increased on M(LPS/IFN-γ) compared to M(M-CSF) and M(IL-4). In contrast, the expression of scavenger receptors such as CD206, CD209, and CD163 was increased on M(IL-4) compared to M(LPS/IFN-γ) (Figures 2B and S1). In addition, we analyzed the relative mRNA expression levels of two transcription factors, *STAT1* and *STAT6* by qRT-PCR. As expected, *STAT1* mRNA expression was only increased in M(LPS/IFN-γ). Increased *STAT6* mRNA expression was observed in M(LPS/IFN-γ) compared to M(M-CSF) and M(IL-4) (Figure 2C). Next, we analyzed cytokines secreted by these macrophages. Cell culture supernatants from M(LPS/IFN-γ) contained higher amounts of the inflammatory cytokines TNF-α, IL-1β, and IL-6 compared to M(M-CSF) and M(IL-4), whereas M(IL-4) produced higher amounts of the chemokine CCL-18, which is in line with previously reported cytokine profiles for these macrophage activation states^29^ (Figure 2D). Lastly, we quantified phagocytosis of *Staphylococcus aureus*-coated beads in M(M-CSF), M(LPS/IFN-γ), and M(IL-4). We observed that M(LPS/IFN-γ) had reduced phagocytosis compared to M(M-CSF) and M(IL-4) (Figure S1). In conclusion, cell-surface markers and soluble mediators confirm that our *ex vivo* generated macrophages are phenotypically representative of resting, inflammatory, and immunosuppressive macrophages.

### M(LPS/IFN-γ) produce increased extracellular proteolytic activity, whereas M(IL-4) have increased cell-associated proteolytic activity

**Figure 3:**
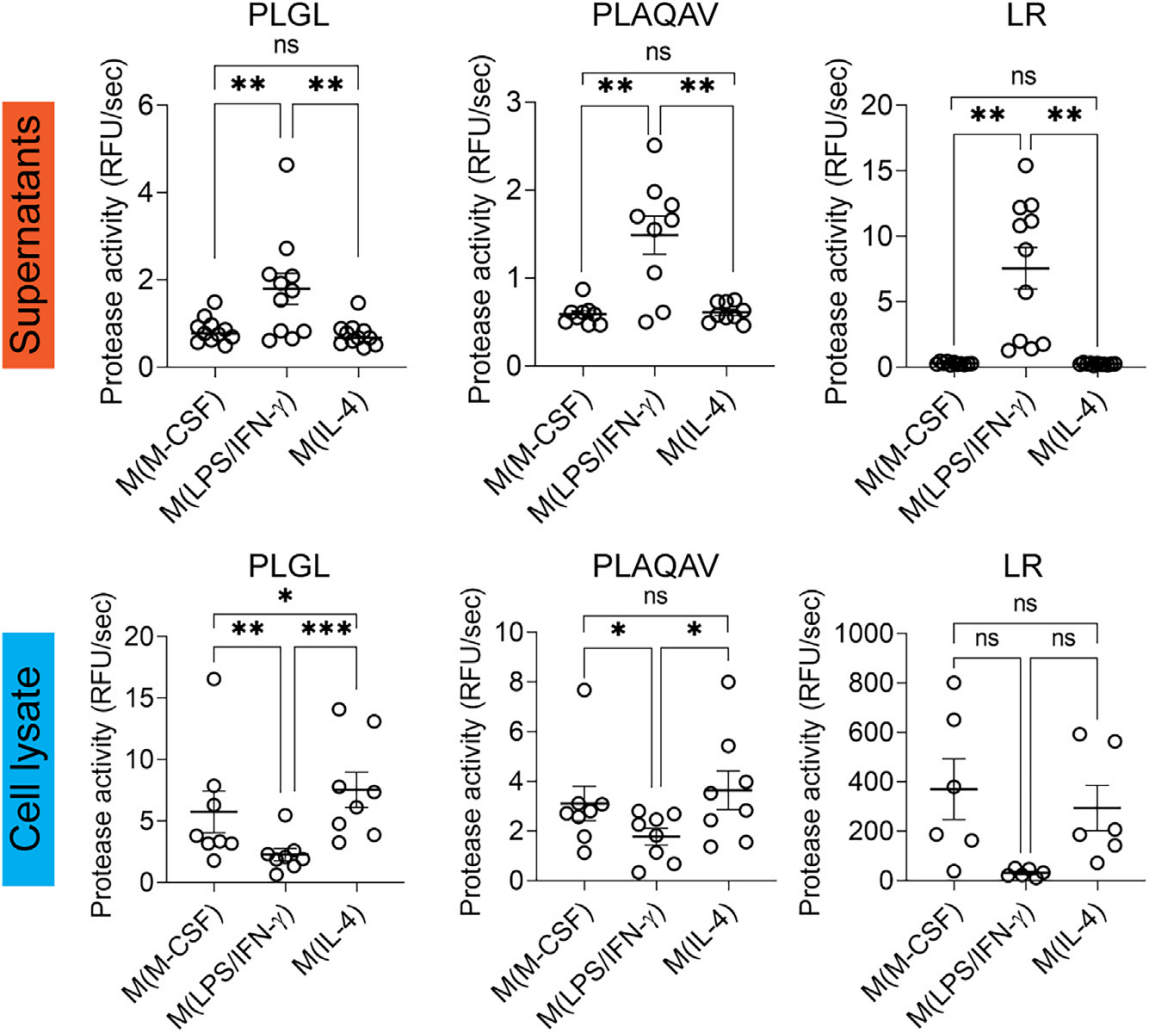
M(LPS/IFN-γ) produce increased extracellular proteolytic activity, whereas M(IL-4) have increased cell-associated proteolytic activity Quantification of the secreted and cell-associated proteolytic activity of M(M-CSF), M(LPS/IFN-γ) and M(IL-4) by substrate-based degradation assays. Mca-PLGL-Dpa-AR-NH2 fluorogenic peptide was used to measure proteolytic MMP and Cathepsin D and E activity. Mca-PLAQAV-Dpa-RSSSR-NH2 fluorogenic peptide was used to measure proteolytic ADAM-9, ADAM-10 and ADAM-17 activity. Z-LR-AMC fluorogenic peptide was used to measure proteolytic cathepsin B, L, and V activity. Each data point represents cells from a different donor (at least *n* = 9 for supernatants and *n* = 6 for cell lysate). Error bars are mean ± SEM. *p* values less than 0.05 were considered significant (∗*p* < 0.05; ∗∗*p* < 0.01; ∗∗∗*p* < 0.001; ns non-significant). See also Figures S4 and S5.

To explore the potential role of proteases produced and secreted by inflammatory and immunosuppressive macrophages, we measured the overall net proteolytic activity using three fluorogenic peptide substrates: PLGL (substrate for MMPs, cathepsin D and cathepsin E), PLAQAV (substrate for TACE, ADAM-9 and ADAM-10) and LR (substrate for cathepsin B, cathepsin L and cathepsin V). We evaluated their proteolysis in macrophage culture supernatants and cell lysates. As expected, for all three substrates, the highest proteolytic activity was found in supernatants of M(LPS/IFN-γ), whereas lower and comparable proteolytic activity was seen in supernatants of M(M-CSF) and M(IL-4) (Figure 3 top panel and S4). In cell lysates, the highest activity was found in M(IL-4) and M(M-CSF) (Figure 3 bottom panel and S5). In particular, the MMP substrate PLGL appeared to undergo significantly increased proteolysis in the M(IL-4) cell lysate. Additionally, by *in situ* gelatin zymography, which allows us to measure gelatinolytic activity, we observed the highest proteolytic activity on the cell surface of M(M-CSF) compared to M(LPS/IFN-γ) and M(IL-4) (Figure S5).

### Proteases and protease inhibitors/activators are increased in cell culture supernatants and cell lysate of M(IL-4) compared to M(LPS/IFN-γ)

**Figure 4:**
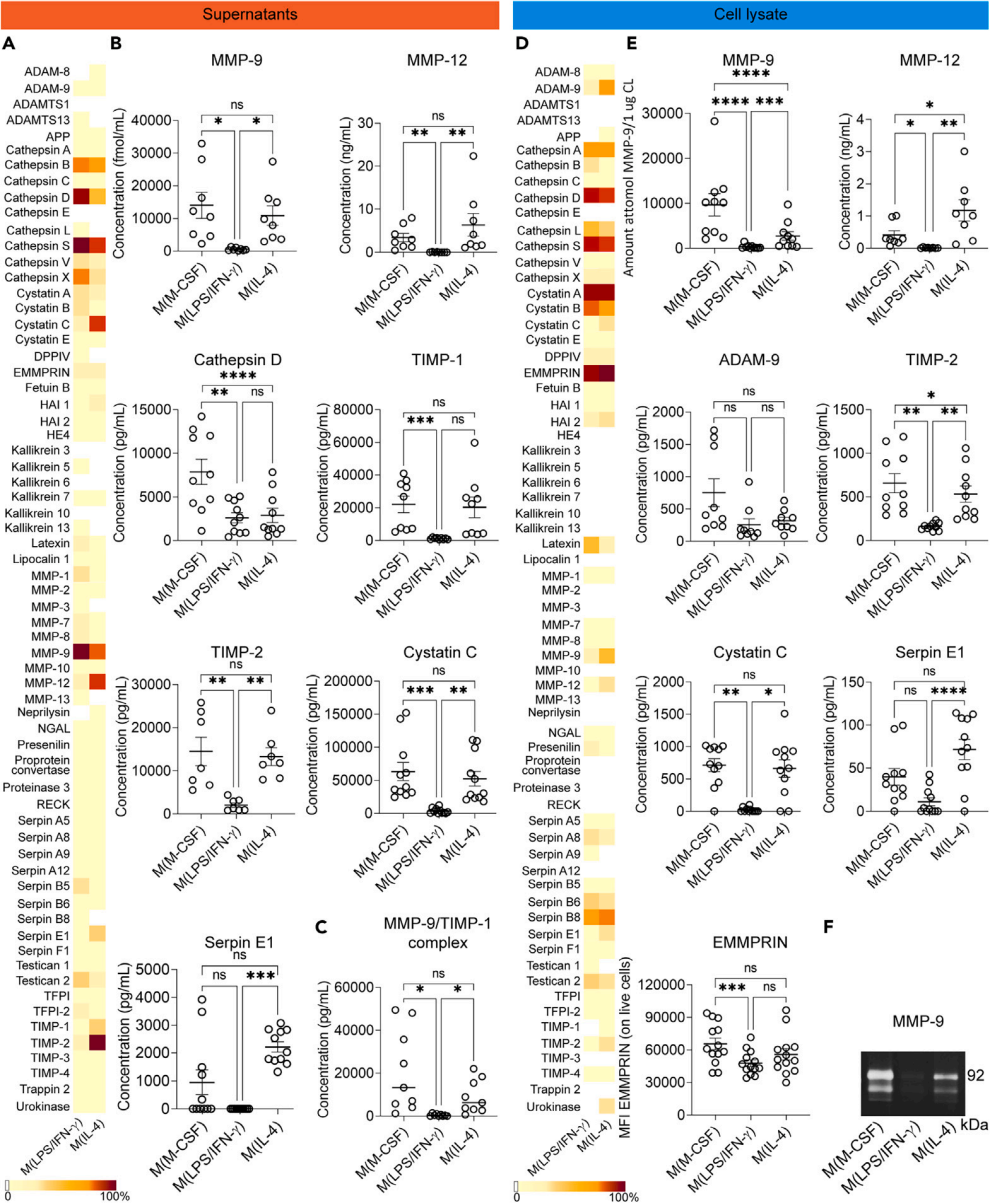
Proteases and protease inhibitors/activators are increased in the cell culture supernatants and cell lysate of M(IL-4) compared to M(LPS/IFN-γ) (A) Protease and protease regulators detected in cell culture supernatants of M(M-CSF), M(LPS/IFN-γ), and M(IL-4) by proteome profiler array analysis. Expression was measured in comparison to the internal array controls and depicted as a scale bar from 0% to 100%. White squares indicate undetectable expression levels. (B) Quantification of secreted proteases and regulators in cell culture supernatants of M(M-CSF), M(LPS/IFN-γ), and M(IL-4) by gelatin *in gel* zymography (for MMP-9 protein levels) and ELISA (for MMP-12, cathepsin D, TIMP-1, TIMP-2, serpin E1, cystatin C). Each data point represents cells from a different donor (at least *n* = 7). Error bars are mean ± SEM. *p* values less than 0.05 were considered significant (∗*p* < 0.05; ∗∗*p* < 0.01; ∗∗∗*p* < 0.001; ∗∗∗∗*p* < 0.0001; ns non-significant). (C) Quantification of secreted MMP-9/TIMP-1 complex levels in cell culture supernatants of M(M-CSF), M(LPS/IFN-γ), and M(IL-4) by ELISA. Each data point represents cells from a different donor (*n* = 9). Error bars are mean ± SEM. *p* values less than 0.05 were considered significant (∗*p* < 0.05; ns non-significant). (D) Analysis of proteases and protease regulators in cell lysates of M(M-CSF), M(LPS/IFN-γ), and M(IL-4), measured using proteome profiler arrays. Expression was measured in comparison to the internal array controls and depicted as a scale bar from 0% to 100%. White squares indicate undetectable expression levels. (E) Quantification of cell-associated proteases and regulators of M(M-CSF), M(LPS/IFN-γ), and M(IL-4) by gelatin *in gel* zymography (for MMP-9 protein levels), ELISA (for MMP-12, ADAM-9, TIMP-2, serpin E1, and cystatin C) and flow cytometry (for EMMPRIN). Each data point represents cells from a different donor (at least *n* = 8). Error bars are mean ± SEM. *p* values less than 0.05 were considered significant (∗*p* < 0.05; ∗∗*p* < 0.01; ∗∗∗*p* < 0.001; ∗∗∗∗*p* < 0.0001; ns non-significant). (F) MMP-9 protein quantification by gelatin *in gel* zymography in the cell lysate of M(M-CSF), M(LPS/IFN-γ), and M(IL-4). See also Figures S2–S5.

As we observed differences in proteolytic activity in cell culture supernatants and cell lysate of M(M-CSF), M(LPS/IFN-γ), and M(IL-4), we further identified proteases and regulatory molecules (i.e., inhibitors and activators) in the samples using a proteome profiler array. (Figures 4A, 4D, S2, and S3). Immunoreactivity for MMP-12 was increased in the cell culture supernatants of M(IL-4), whereas immunoreactivity for MMP-9, aspartyl protease cathepsin D, and cysteine protease cathepsin X was increased in M(LPS/IFN-γ). As for the protease regulators, the MMP inhibitors TIMP-1 and TIMP-2, as well as the cysteine protease inhibitor cystatin C and the serine protease inhibitor serpin E1 (also known as PAI-1), were increased in culture supernatants of M(IL-4) (Figures 4A and S2). We verified these differences in protein levels by gelatin *in gel* zymography, ELISA, or flow cytometry in macrophages originating from several donors and included resting macrophages (M(M-CSF)) in our analysis. While the results for MMP-12, TIMP-1, TIMP-2, cystatin C, and serpin E1 could be confirmed, MMP-9 and cathepsin D were not (Figure 4B). Quantification of MMP-9 protein levels by gelatin *in gel* zymography and Western blot analysis showed a 20-fold increase of MMP-9 (monomeric proteoform) in the cell culture supernatants of M(IL-4) compared to M(LPS/IFN-γ) (Figures 4B and S4). As for the quantification of cathepsin D, ELISA analysis showed that M(M-CSF) are the main secretors of cathepsin D, whereas M(LPS/IFN-γ) and M(IL-4) secrete lower and comparable levels (Figure 4B). These findings are particularly interesting given the recent discovery that several cathepsins, although often considered lysosomal proteases implicated in protein catabolism and bacterial killing, are now being found in the extracellular environment where they have additional functions.^31^ In addition, M(IL-4) secreted increased levels of cysteine protease inhibitor cystatin C and the serine protease inhibitor serpin E1 (Figure 4B).

In conclusion for cell culture supernatants, it appears that M(IL-4) secrete the highest levels of proteases compared to M(LPS/IFN-γ) but also secrete higher levels of protease inhibitors leading to increased levels of protease-inhibitor complexes. For example, the metalloproteinases MMP-9 and MMP-12 are increasingly secreted by M(IL-4) compared to M(LPS/IFN-γ), but also considerably higher levels of their inhibitors TIMP-1 and TIMP-2 (Figure 4B). Indeed, we observed increased levels of the MMP-9/TIMP-1 complex in supernatants of M(IL-4) compared to M(LPS/IFN-γ) (Figure 4C).

Cell lysate of M(IL-4) contained higher levels of MMP-9, MMP-12, ADAM-9, and uPA, and the inhibitors TIMP-2, cystatin C, serpin E1 and the MMP activator EMMPRIN compared to M(LPS/IFN-γ). Furthermore, the protease inhibitor latexin was increased in M(LPS/IFN-γ) (Figures 4D and S3). Again, we confirmed these differences by gelatin *in gel* zymography, ELISA, or flow cytometry in macrophages originating from different donors and also included resting macrophages (M(M-CSF)) in our analysis. Gelatin *in gel* zymography and Western blot analysis revealed a 10-fold increase of MMP-9 (monomeric proteoform) in cell lysate of M(IL-4) compared to M(LPS/IFN-γ) (Figures 4E, 4F, and S5), thereby confirming the proteome profiler data. Moreover, the highest amount of cell-associated MMP-9 was found in M(M-CSF). Interestingly, gelatin *in gel* zymography showed an additional band (84 kDa) underneath the 92 kDa band of proMMP-9, suggesting the presence of either activated MMP-9 or another glycosylated form of MMP-9 present in M(IL-4) (Figures 4F and S5). Both protein quantification by Western blot analysis and ELISA confirmed that M(IL-4) contained the highest levels of cell-associated MMP-12 as compared to M(LPS/IFN-γ) and M(M-CSF) (Figures 4E and S5). In line with the proteome profiler array, we also observed an increased, though not significant, presence of ADAM-9 in the cell lysate of M(IL-4) (Figure 4E). Regarding protease regulators, we observed an increase in cystatin C and serpin E1 in the cell lysate of M(IL-4) compared to M(LPS/IFN-γ). Cell-associated TIMP-2 was significantly elevated in M(M-CSF) and M(IL-4) compared to M(LPS/IFN-γ). (Figure 4E). Given the importance of TIMP-1 in protease regulation and its alterations shown in cell culture supernatants (Figure 4B), we also investigated cell-associated TIMP-1 levels. A 4-fold increase in TIMP-1 was observed in the cell lysate of M(IL-4) compared to M(LPS/IFN-γ). Interestingly, the amount of MMP-9 complexed by TIMP-1 was similar between M(LPS/IFN-γ) and M(IL-4) (Figure S5). Flow cytometric analysis of EMMPRIN showed a slightly increased, although not significant, expression on the cell surface of M(IL-4) compared to M(LPS/IFN-γ) (Figure 4E).

### Analysis of matrix metalloproteinases and their inhibitors in macrophage cell compartments

**Figure 5:**
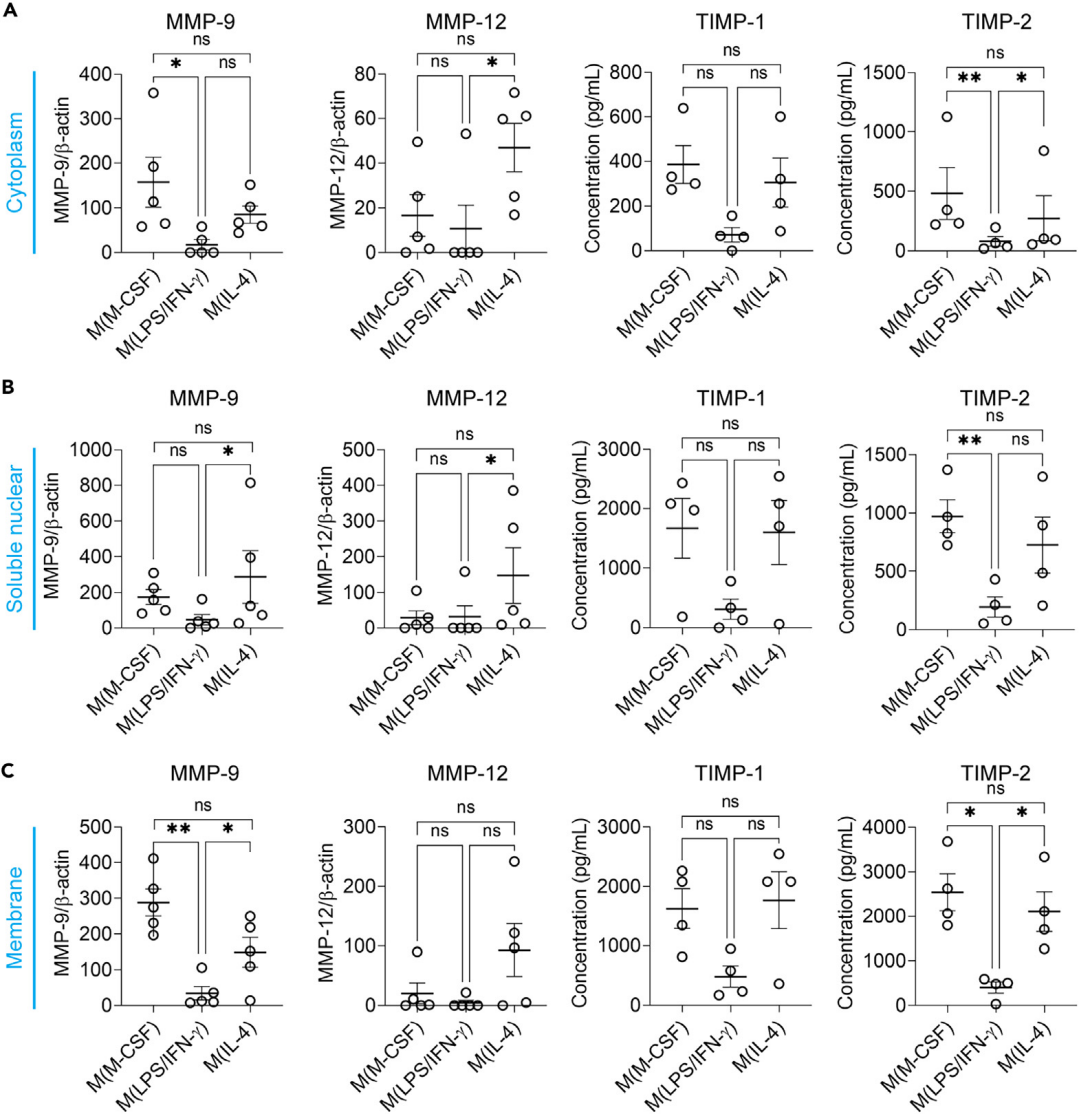
Analysis of MMPs and their inhibitors in macrophage cell compartments Cellular protein fractions of M(M-CSF), M(LPS/IFN-γ) and M(IL-4), including cytoskeleton, cytoplasm, soluble nuclear, chromatin-bound and membrane protein fractions were analyzed by immunoblotting for MMP-9 and MMP-12, and by ELISA for TIMP-1 and TIMP-2. (A) Quantification of total MMP-9 (*n* = 5), total MMP-12 (*n* = 5), TIMP-1 (*n* = 4) and TIMP-2 (*n* = 4) in the cytoplasm protein fraction of M(M-CSF), M(LPS/IFN-γ) and M(IL-4), determined by Western blot analysis (data were normalized to β-actin) and ELISA. (B) Quantification of total MMP-9 (*n* = 5), total MMP-12 (*n* = 5), TIMP-1 (*n* = 4) and TIMP-2 (*n* = 4) in the soluble nuclear protein fraction of M(M-CSF), M(LPS/IFN-γ) and M(IL-4), determined by Western blot analysis (data were normalized to β-actin) and ELISA. (C) Quantification of total MMP-9 (*n* = 5), total MMP-12 (*n* = 5), TIMP-1 (*n* = 4) and TIMP-2 (*n* = 4) in the membrane protein fraction of M(M-CSF), M(LPS/IFN-γ) and M(IL-4), determined by Western blot analysis (data were normalized to β-actin) and ELISA. Each data point represents cells from a different donor. Error bars are mean ± SEM. *p* values less than 0.05 were considered significant (∗*p* < 0.05; ∗∗*p* < 0.01; ns non-significant). See also Figure S6.

Recent studies point toward the intracellular localization and function of several known extracellular proteases.^18^^,^^19^ We aimed to localize proteases and regulators within the cell, focusing on MMP-9, MMP-12, TIMP-1, and TIMP-2, based on their increased presence in the cell lysate of M(IL-4) compared to M(LPS/IFN-γ) and their lesser-known intracellular roles (Figures 5 and S6). Given that macrophages secrete A2M, a broad-spectrum protease inhibitor no included in the array, and express A2M receptors, we also included A2M in our analysis^32^ (Figure S6). Cell fractionation followed by ELISA and Western blot revealed that the cytoplasm protein fraction of M(IL-4) contained significantly more MMP-12 and TIMP-2 compared to M(LPS/IFN-γ), whereas MMP-9 and TIMP-2 were not significantly increased (Figures 5A and S6). Interestingly, the soluble nuclear protein fraction of M(IL-4) showed a significant increase in MMP-9, MMP-12 and A2M compared to M(LPS/IFN-γ) (Figures 5B and S6). Furthermore, MMP-9, TIMP-2 and A2M were increased in the membrane protein fraction of M(IL-4) compared to M(LPS/IFN-γ) (Figures 5C and S6). The cytoskeletal and chromatin-bound protein fractions showed similar or undetectable protein levels of MMP-9, MMP-12, TIMP-1, TIMP-2 and A2M between M(M-CSF), M(LPS/IFN-γ) and M(IL-4) (Figure S6).

### Cellular entry may dictate intracellular matrix metalloproteinase-9 localization in M(IL-4)

**Figure 6:**
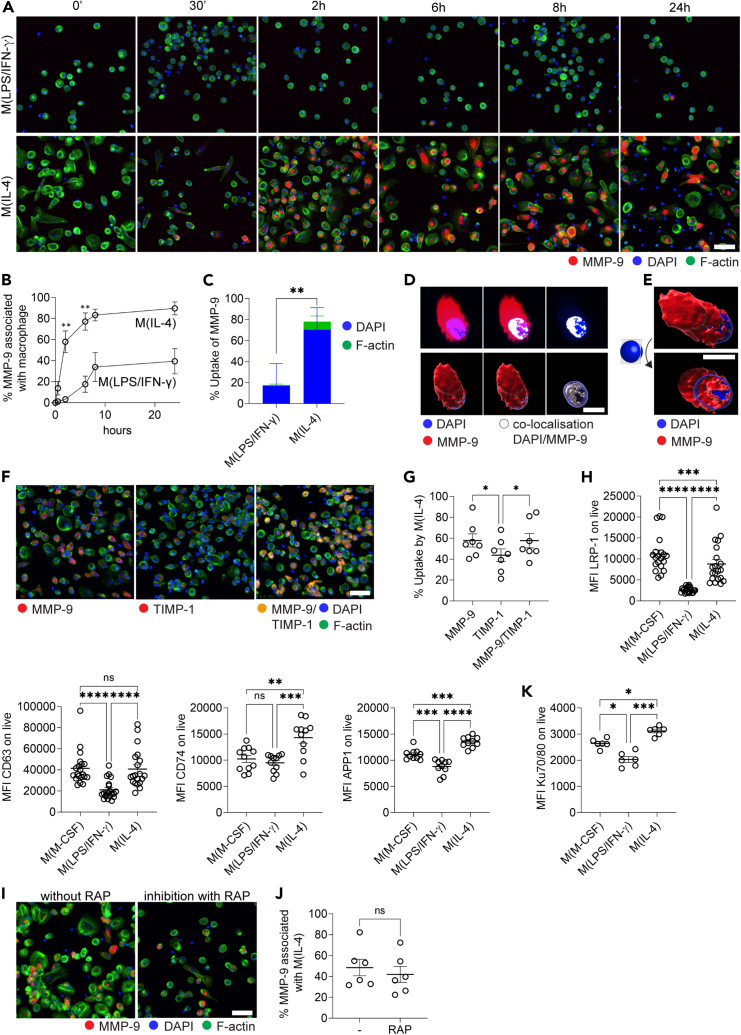
Cellular entry may dictate intracellular MMP-9 localization in M(IL-4) (A) Representative immunofluorescent images of M(LPS/IFN-γ) and M(IL-4) over time (0 min–24 h) upon incubation with exogenous fluorescently labeled recombinant human MMP-9 (red). The structure of cells is visualized by F-actin staining (Phalloidin, green) and nuclei are visualized by Hoechst 33342 staining (DAPI, blue). Images are representative of 8 different donors. The scale bar represents 50 μm. (B) Quantification of MMP-9 uptake over time by M(LPS/IFN-γ) and M(IL-4) (*n* = 8). Error bars are mean ± SEM. *p* values less than 0.05 were considered significant (∗∗*p* < 0.01). (C) Quantification of immunofluorescent images of M(LPS/IFN-γ) and M(IL-4) after 6 h of incubation with fluorescently labeled MMP-9. The blue bar shows MMP-9 co-localization with the nucleus, and the green bar shows MMP-9 co-localization with F-actin (in the absence of nuclear co-localization) (*n* = 8). Error bars are mean ± SEM. *p* values less than 0.05 were considered significant (∗∗*p* < 0.01). (D) Representative confocal images of M(IL-4) incubated with fluorescently labeled recombinant human MMP-9. The nucleus is shown in blue (DAPI) and MMP-9 is shown in red. Regions of MMP-9/DAPI colocalization are shown in white. The top panel shows confocal images; the bottom panel shows a surface depiction of the confocal images. The scale bar represents 10 μm. (E) Confocal images of M(IL-4) incubated with exogenous fluorescently labeled recombinant human MMP-9. The nucleus is shown in blue (DAPI) and MMP-9 is shown in red. The top panel shows a surface depiction of MMP-9. The bottom panel shows a coronal cross section (XY plane) of the surface depiction of MMP-9. The scale bar represents 10 μm. (F) Representative immunofluorescent images of M(IL-4) incubated with exogenous fluorescently labeled recombinant MMP-9 (red, left panel), TIMP-1 (red, middle panel) and MMP-9/TIMP-1 complex (orange, right panel). The structure of cells is visualized by F-actin staining (Phalloidin, green) and nuclei are visualized by Hoechst 33342 staining (DAPI, blue). Images are representative of 7 different donors. The scale bar represents 50 μm. (G) Average uptake (between 30 min and 6 h) of exogenous fluorescently labeled recombinant MMP-9, TIMP-1, and MMP-9/TIMP-1 complex. Each data point represents cells from a different donor (*n* = 7). Error bars are mean ± SEM. *p* values less than 0.05 were considered significant (∗*p* < 0.05). (H) Flow cytometry analysis of LRP-1, CD63, CD74, and APP1 on M(M-CSF), M(LPS/IFN-γ) and M(IL-4). Each data point represents cells from a different donor (at least *n* = 9). Error bars are mean ± SEM. *p*-values less than 0.05 were considered significant (∗∗*p* < 0.01; ∗∗∗*p* < 0.001; ∗∗∗∗*p* < 0.0001; ns non-significant). (I) Representative immunofluorescent images of M(IL-4) incubated with exogenous fluorescently labeled recombinant MMP-9 (red) in the presence of RAP (LRP-1 inhibitor). Structure of cells is visualized by F-actin staining (Phalloidin, green) and nuclei are visualized by Hoechst 33342 staining (DAPI, blue). Images are representative of 6 different donors. The scale bar represents 50 μm. (J) Average uptake (between 30 min and 6 h) of exogenous fluorescently labeled recombinant MMP-9 in the presence of RAP (LRP-1 inhibitor). Each data point represents cells from a different donor (*n* = 6). Error bars are mean ± SEM. *p* values less than 0.05 were considered significant (ns non-significant). (K) Flow cytometry analysis of translocating protein Ku70/80 on M(M-CSF), M(LPS/IFN-γ), and M(IL-4). Each data point represents cells from a different donor (*n* = 6). Error bars are mean ± SEM. *p*-values less than 0.05 were considered significant (∗*p* < 0.05; ∗∗∗*p* < 0.001). See also Figure S7.

Given the identified intracellular presence of MMP-9 and MMP-12 in M(IL-4), we next addressed the mechanisms by which they could reach the respective subcellular compartments. Since, to our knowledge, the presence of intracellular MMP-9 in (resolving) macrophages has never been described, we decided to study this protein in more detail. It has been reported that secreted MMPs may re-enter cells by endocytosis or translocating proteins to move across membranes.^18^^,^^19^ To investigate this, we administered exogenous fluorescently labeled MMP-9 to M(LPS/IFN-γ) and M(IL-4) and followed its uptake over time. We observed a significantly increased uptake of MMP-9 by M(IL-4) compared to M(LPS/IFN-γ) (Figures 6A and 6B). To confirm the specificity of MMP-9 intracellular uptake, we also followed the uptake of fluorescently labeled BSA, which did not cross into the intracellular compartments of the macrophages (Figure S7). Interestingly, the MMP-9 signal clearly co-localized with the cell nucleus following 2 h of MMP-9 uptake (Figures 6C–6E and S7), suggesting that after uptake, MMP-9 can relocate to the cell nucleus. Given that in our previous experiment (Figure 5), we observed intracellular increases of the MMP-9 inhibitor TIMP-1, we next tracked the cellular localization of TIMP-1 and its complex with MMP-9. We measured the cellular uptake of fluorescently labeled TIMP-1 and fluorescently labeled MMP-9/TIMP-1 complex in M(LPS/IFN-γ) and M(IL-4). Again, we observed that M(IL-4) showed a significantly increased uptake of TIMP-1 and MMP-9/TIMP-1 complexes compared to M(LPS/IFN-γ) (Figures 6F and S7). Interestingly, the uptake of free TIMP-1 was significantly slower compared to the uptake of free MMP-9 in M(IL-4). However, when TIMP-1 formed a complex with MMP-9, the uptake increased and was comparable to the amount of uptake of free MMP-9, indicating that complex formation with MMP-9 facilitates the uptake of TIMP-1 (Figures 6F, 6G, and S7). As several membrane receptors are known to bind MMP-9 and/or TIMP-1 and mediate their endocytosis,^33^^,^^34^^,^^35^ we next tried to determine the involved membrane receptor in M(IL-4). The scavenger receptors LRP-1 and LRP-2, well-known receptors for the uptake and endocytosis of MMP-9,^36^^,^^37^^,^^38^ were highly expressed on M(IL-4) compared to M(LPS/IFN-γ), suggesting that these receptors could mediate the uptake of free and complexed MMP-9 in M(IL-4) (Figures 6H and S7). When blocking LRP-1-mediated endocytosis with RAP, a molecule that binds tightly to LRP-1 and antagonizes ligand binding, we observed only a minor reduction in MMP-9 uptake by M(IL-4), suggesting other mechanisms must contribute to the uptake of MMP-9 (Figures 6I, 6J, and S7). Indeed, CD63, CD74, and APP, three recently discovered TIMP-1-binding receptors,^39^^,^^40^^,^^41^ were also highly expressed on M(IL-4) compared to M(LPS/IFN-γ) (Figure 6H). Finally, CD44, a glycoprotein receptor critical for MMP-9-mediated cell migration,^42^^,^^43^ was equally expressed on all three macrophage types (Figure S7). Interestingly, M(IL-4) showed an increased expression of Ku70, a DNA repair protein, compared to (LPS/IFN-γ), suggesting that MMP-9 may bind to translocating proteins at the cell surface of M(IL-4) to move across the cell membrane (Figure 6K). Another mechanism by which MMP-9 and TIMP-1 may be localized to the nucleus is through the possession of a typical nuclear localization sequence.^18^^,^^19^ While several MMPs^44^^,^^45^^,^^46^^,^^47^ have been reported to contain such sequences, MMP-9 has not. Therefore, we evaluated the presence of putative nuclear localization sequences using existing prediction tools that look for targeting signal features, sequence-based features, or annotation-based features. The DeepLoc 2.0 server,^48^ which predicts eukaryotic protein subcellular localization using deep learning, indicated that the selected proteases (MMP-9 and MMP-12) and protease inhibitors (TIMP-1, TIMP-2 and A2M) are mainly secreted proteins. Indeed, all of these proteins have a standard N-terminal signal peptide that should result in protein secretion (SignalP 6.0 prediction tool^49^). However, nuclear localization sequences were not found at the protein N-terminus. The classical pathway for proteins with a molecular weight above 40 kDa to traffic to the nucleus involves binding to importins via a nuclear localization sequence, typically composed of one or two clusters of 4–5 positively charged amino acids (arginine, lysine).^50^ When searching the NLSdb database^51^ for nuclear localization sequences and nuclear export signals, no existing sequences were found in the selected proteases and inhibitors. However, using a different nuclear localization sequence predictor tool, INSP,^52^ a nuclear localization sequence was predicted for MMP-9, TIMP-2 and A2M (Table 1). This suggests that the nuclear uptake of MMP-9 may be facilitated through this sequence. Further *in vitro* validation is needed to investigate how MMP-9 mediates its uptake in M(IL-4).

**Table 1:** Predicated nuclear localization sequence (NLS) by using the INSP tool

Protease/Inhibitor	Predicted NLS (INSP^52^)	Domain
MMP-9	^450^EPRPP^454^	O-glycosylated
MMP-12	–	–
TIMP-1	–	–
TIMP-2	^194^IKRSD^198^	C-terminal
A2M	^680^NSKIRKP^686^	Bait region

## Discussion

Macrophages play an important role in the initiation and resolution of inflammation. Their remarkable plasticity allows them to acquire functionally distinct phenotypes.^53^ However, little is known about how macrophage proteases change with macrophage plasticity or how these changes may shape macrophage function and contribute to their phenotype.

One technological hurdle in profiling proteolytic activity is the common use of high concentrations of FBS/FCS in cell culture conditions, which contain high quantities of bovine proteases and their inhibitors. To address this, we established an *in vitro* approach to study macrophage proteases in the absence of exogenous proteases and inhibitors by using a serum replacement for the final 24 h of macrophage culture. We validated the obtained macrophages (M(M-CSF), M(LPS/IFN-γ) and M(IL-4)) based on cell shape, cell-surface markers, gene expression, and cytokine profiles. All parameters, except for *STAT6* mRNA levels,^54^ matched the typical profile of M(M-CSF), M(LPS/IFN-γ) and M(IL-4). Activation of signal transduction cascades resulting in the activation of STATs is a rapid upstream process, which implies that this cascade might have already been fully triggered and subsided after 3 days of IL-4 stimulation when measuring *STAT6* expression levels. Indeed, Venkataraman et al. observed that *STAT6* DNA binding activity was present at 14 h following IL-4 stimulation but was absent after 28 h.^55^ Importantly, all other biochemical and marker analyses for macrophage differentiation confirm the expected phenotypes under these conditions.^53^ We conducted a side-by-side comparison of the proteases, inhibitors, and net proteolytic activity produced by human M(M-CSF), M(LPS/IFN-γ) and M(IL-4). We demonstrate that M(LPS/IFN-γ) display a higher extracellular proteolytic potential compared to M(IL-4). Previous studies have reported that pro-inflammatory macrophages release a set of proteases contributing to the destruction of the extracellular matrix, cell migration, and cytokine/chemokine activation.^56^^,^^57^ Importantly, our results highlight a different aspect: while M(IL-4) secrete lower amounts of active proteases, they have increased levels of active cell-associated proteases compared to M(LPS/IFN-γ). Furthermore, we observed that M(IL-4) contain increased levels of proteases and inhibitors in their cell lysate compared to M(LPS/IFN-γ). Although proteases in the secretory pathway or present on the cell membrane may partially account for this observation, we demonstrated for a selection of proteases and inhibitors that these are also present in specific cellular organelles.

While alternative intracellular functions of MMPs, including their nuclear localization, have been previously reported,^47^^,^^58^^,^^59^^,^^60^^,^^61^^,^^62^ research on nuclear MMP-9 has been limited. Here, we report that, in addition to MMP-12, MMP-9 is also increasingly present in the nuclear fraction of M(IL-4) compared to M(LPS/IFN-γ). This finding is highly relevant as it implies that macrophages can reallocate proteases to different cellular compartments depending on their phenotype. It has been reported that MMP-12 can target substrates both transcriptionally and post-translationally, potentially influencing the macrophage immune response.^20^ Specifically, MMP-12 secreted by macrophages can be taken up by virus-infected cells, where it acts as a transcription factor to induce IFN-α secretion and host protection,^20^ but can also cleave IFN-α leading to a negative feedback mechanism. Based on the similarities between MMP-2 and MMP-9, nuclear localization and activity of MMP-9 have been tied to research on MMP-2. In cerebral ischemia models, MMP-2 cleaves PARP1 and XRCC1, two DNA repair enzymes, *in vitro* and *in vivo* to induce neuronal apoptosis.^59^^,^^60^ However, unlike MMP-2, a direct interaction between MMP-9 and PARP1 or XRCC1 has not yet been shown.^60^ Additionally, our flow cytometry and immunofluorescence data do not indicate that the observed intracellular presence of MMP-9 and increased proteolytic activity induce apoptosis in M(IL-4). Other intracellular functions, both proteolytic and non-proteolytic, for MMP-9, have not been reported. However, we may speculate that similar roles observed for other nuclear proteases might be assigned to the nuclear presence of MMP-9 in M(IL-4). An important example could come from the observation of nuclear MMP-14 in murine macrophages. It was shown that over 100 genes, mostly linked to the immune response, are affected by nuclear MMP-14 independent of its protease activity. In particular, nuclear MMP-14 triggers the expression and activation of a phosphoinositide 3-kinase δ (PI3Kδ)/Akt/GSK3β signaling cascade, allowing macrophages to transition from a defense to a resolution response.^21^

The biological mechanisms that dictate the subcellular localization of MMPs are far from understood. Here, we show a phenotype-dependent uptake of MMP-9 by macrophages. Specifically, our findings show that exogenous MMP-9 can be efficiently taken up by M(IL-4) compared to M(LPS/IFN-γ) and is then directed toward the nucleus. The uptake of TIMP-1 was significantly slower than MMP-9 but was restored when in complex with MMP-9, suggesting the uptake is mainly dependent on MMP-9. Furthermore, we showed that M(IL-4) had increased expression levels of several known cell-surface interaction partners for MMP-9 and TIMP-1 compared to M(LPS/IFN-γ). This further strengthens the increased capacity of M(IL-4) to tether MMP-9/TIMP-1 to the cell surface, and potentially facilitate their uptake. A major increase was seen in the scavenger receptor LRP-1. Free and complex MMP-9 are reported to bind LRP-1 via its hemopexin domain to facilitate its internalization.^36^^,^^38^ However, when blocking this receptor with RAP, we observed only a minor decrease in MMP-9 uptake. This suggests that either other mechanisms of uptake must be in place or that the recycling rate of LRP-1, and the subsequent loss of RAP inhibition, is too high to observe any effect. Furthermore, binding to LRP-1 normally results in ligand catabolism, a mechanism often used to reduce the extracellular concentration of proteases, cytokines, and other ligands.^36^ Consequently, how MMP-9 escapes this LRP-1-mediated catabolism to reach the nucleus remains unclear. It is suggested that MMP-9 can also bind to translocating proteins to move across membranes and enter the nucleus, such as nuclear protein Ku, RNA, and RNA binding proteins.^63^^,^^64^ Interestingly, we observed an increased presence of Ku70 on the cell membrane of M(IL-4) compared to M(LPS/IFN-γ). Another mechanism for nuclear entry is through the possession of a nuclear localization sequence.^18^^,^^19^^,^^44^^,^^45^^,^^46^ Although MMP-9 and TIMP-1 have been reported in the nucleus of cells, no nuclear localization sequence for either has been identified, except for the database INSP, which predicted a nuclear localization sequence in MMP-9.

In summary, our study revealed that M(LPS/IFN-γ) exhibit increased extracellular proteolytic activity compared to M(IL-4). In contrast, IL-4 stimulation increased cell-associated proteolytic activity, particularly for MMPs. We observed that proteases and inhibitors are present within M(IL-4) and in specific organelles, such as the nucleus. We report an increased presence of MMP-9 in the nucleus of M(IL-4), possibly mediated through the uptake of MMP-9 from the extracellular environment, a process not observed in M(LPS/IFN-γ). It remains to be determined whether the observed changes in proteolytic activity and localization also occur in other macrophage phenotypes, such as M(GM-CSF), M(LPS) and M(IL-13). In conclusion, we introduce an *in vitro* approach governing the phenotype-dependent regulation of proteolysis in human primary macrophages. Further investigation to elucidate the functional implications of this mechanism promises intriguing findings with major relevance in the field of inflammation research and macrophage biology.

### Limitations of the study

A limitation of our study is that it starts from a proteome profiler array, restricting our analysis to the proteases and protease regulators included in the array. Consequently, we miss some key proteases essential for macrophage function such as MT1-MMP/MMP-14. Additionally, our study is based on an *in vitro* model, which does not fully replicate the *in vivo* environment. Future studies could build upon this foundation to investigate the *in vivo* contexts further. In our *in vitro* model, we utilize human macrophages derived from blood monocytes donated by healthy volunteers (through the Red Cross). The donors are a mix of male and female volunteers, but we do not know the sex of each donor individually.

## Resource availability

### Lead contact

Further information and requests for resources and reagents should be directed to and will be fulfilled by the lead contact, Jennifer Vandooren (jennifer.vandooren@kuleuven.be).

### Materials availability

This study did not generate new unique materials or reagents.

### Data and code availability

• This article does not report novel RNA-seq data, nucleotide sequencing-associated datasets, proteomics, peptidomics, metabolomics, structures of biological macromolecules, or small-molecule crystallography.
• This article does not report the original code.
• Any additional information about the data reported in this article will be shared by the lead contact upon request.

## Acknowledgments

This work was supported by grants from the Belgian 10.13039/501100006401Charcot Foundation (2020, 2021). E.B. and A.D.V received an 10.13039/501100003130FWO fellowship for fundamental research (11H9123N and 11H9125N; 11K0722N and 11K0724N respectively). K.A. received an FWO-SB fellowship for strategic research (1S75320N). Graphical abstract and Figure 2A were created with BioRender.com.

## Author contributions

Conceptualization, E.B., J.V., and P.M.; methodology, E.B., K.A., A.D.V., B.M., A.K., P.M., and J.V.; validation, E.B., T.M., and J.V.; formal analysis, E.B., A.D.V., and J.V.; investigation, E.B, T.M., E.K., and J.V.; resources, D.H. and A.K.; writing – original draft, E.B and J.V.; writing – review and editing, E.B., K.A., A.D.V, B.M., D.H., T.M., E.K., A.K., L.D.S, P.M., and J.V.; visualization, E.B., A.D.V., and J.V.; supervision, P.M. and J.V.; project administration, E.B. and J.V.; funding acquisition, P.M. and J.V.

## Declaration of interests

The authors declare no competing interests.

## Declaration of generative AI and AI-assisted technologies in the writing process

During the final revision of this work the authors used Copilot as a language assistant to improve readability and language of the work.

## STAR★Methods

### Key resources table

**Table:** 

REAGENT or RESOURCE	SOURCE	IDENTIFIER
**Antibodies**		

Anti-CD80 BV510 (clone 2D10)	Biolegend	Cat # 305233; RRID: AB_2687023
Anti-CD86 PE-Cy7 (clone BU63)	Biolegend	Cat # 374209; RRID: AB_2728391
Anti-CD206 BV786 (clone 19.2)	BD Bioscience	Cat # 740999; RRID: AB_2740622
Anti-CD209 BV421 (clone 9E98A)	Biolegend	Cat # 330117; RRID: AB_2734323
Anti-HLA-DR APC (clone L243)	Biolegend	Cat # 307622; RRID: AB_493177
Anti-CD163 PE (clone GHI/61)	Biolegend	Cat # 333606; RRID: AB_1134002
Anti-LRP-1 PE (clone A2MR-alpha-2)	Invitrogen	Cat # 12-0919-42; RRID: AB_2572571
Anti-LRP-2 Alexa Fluor 647 (clone 545606)	R&D Systems	Cat # FAB9578R; RRID: AB_3654045
Anti-EMMPRIN Alexa Fluor 488 (clone HIM8	Biolegend	Cat # 306207; RRID: AB_528739
Anti-CD44 APC/Fire 450 (clone BJ18)	Biolegend	Cat # 338818; RRID: AB_2716003
Anti-APP1 Alexa Fluor 488 (clone 22C11)	Merck	Cat # MAB348A4; RRID: AB_11213747
Anti-CD63 APC (clone H5C6)	Biolegend	Cat # 353008; RRID: AB_10916521
Anti-CD74 Superbright 436 (clone 5–329)	Invitrogen	Cat # 62-0748-42; RRID: AB_2784817
Anti-Ku70/80 PE (clone KU729)	Neobiotechnologies	Cat # 2547-MSM1-PE-100T
Anti-Human MMP-9	R&D systems	Cat # AB911; RRID: AB_354377
Anti-Human MMP-12	R&D systems	Cat # AF917; RRID: AB_3644613
Anti-Human ADAM-9	R&D systems	Cat # DY939
Anti-SP1	Cell Signaling	Cat # 5931; RRID: AB_10621245
Anti-β-actin	Proteintech	Cat # 20536-1; RRID: AB_10700003
Anti-H3	Cell Signaling	Cat # 9715; RRID: AB_331563
Anti-CD44	Cell Signaling	Cat # 3570; RRID: AB_2076465
Anti-Human HSP90	R&D systems	Cat # MAB3286; RRID: AB_2121072
Anti-TIMP-1	Cell Signaling	Cat # 8946S; RRID: AB_10891805
Anti-A2M	R&D Systems	Cat # AF1938; RRID: AB_2221437
Anti-goat IgG HRP-conjugated antibody solution	Vector Labs	Cat # PI-9500; RRID: AB_2336124
Anti-rabbit IgG HRP-conjugated antibody solution	Jackson Immunoresearch	Cat # 711-035-152; RRID: AB_10015282
Anti-mouse IgG HRP-conjugated antibody solution	Jackson ImmunoResearch	Cat # 115-035-071; RRID: AB_2338506

**Biological samples**		

Primary cell cultures: human primary macrophages	Blood Transfusion Center of the Belgian Red Cross, Mechelen, Belgium	RKOV_19006 (Red Cross Biobank)

**Chemicals, peptides, and recombinant proteins**		

Pancoll Human	Pan Biotech	Cat # P04-601000
RPMI 1640 GlutaMAX™ medium	ThermoFisher Scientific	Cat # 61870036
Fetal bovine serum	Sigma-Aldrich	Cat # F7524
Penicillin-Streptomycin (5,000 U/mL)	ThermoFisher Scientific	Cat # 15070063
Human M-CSF/CSF-1 recombinant protein	PeproTech	Cat # 300-25
LPS from Escherichia coli O111:B4	Sigma-Aldrich	Cat # L4391
Human IFN-γ recombinant protein	PeproTech	Cat # 300-02
Human IL-4 recombinant protein	PeproTech	Cat # 200-04
KnockOut™ Serum Replacement	ThermoFisher Scientific	Cat # 10828028
Human fragment crystallizable receptor (FcR) blocking reagent	Miltenyi Biotec	Cat # 130059901
Fixable Viability Stain 620	BD Biosciences	Cat # 564996
Zombie Aqua	Biolegend	Cat # 423102
pHrodo Red S. aureus bioparticles	Invitrogen	Cat # A10010
Hank’s balanced salt solution (HBSS)	ThermoFisher Scientific	Cat # 14065-049
NaHCO_3_	Honeywell Riedel-de-Haën	Cat # 13433
BSA (Albumine V Fraction, protease free)	Carl Roth	Cat # T8444.4
32% paraformaldehyde	Thermo Fisher Scientific	Cat # 047377.9M
CellMask™ Green Actin Tracking Stain	Invitrogen	Cat # A57243
CellMask™ Deep Red Actin Tracking Stain	Invitrogen	Cat # A57245
Hoechst 33342	Invitrogen	Cat # 62249
ProLong diamond mounting medium	ThermoFisher Scientific	Cat # P36961
Alexa Fluor™ 546 Antibody Labeling Kit	ThermoFisher Scientific	Cat # A20183
Alexa Fluor™ 647 Antibody Labeling Kit	ThermoFisher Scientific	Cat # A20186
RAP Recombinant Human LRPAP Protein, CF	R&D Systems	Cat # 4296-LR
RNeasy mini kit	Qiagen	Cat # NC9677589
MultiScribe Reverse Transcriptase	Invitrogen	Cat # 4311235
Random primers	Invitrogen	Cat # 4319979
Mca-PLGL-Dpa-AR-NH2	R&D Systems	Cat # ES001
Mca-PLAQAV-Dpa-RSSSR-NH2	R&D Systems	Cat # ES003
Z-LR-AMC	R&D Systems	Cat # ES008
EDTA	VWR	Cat # 20302.260
Pepstatin A	R&D Systems	Cat # 1190
E 64	R&D Systems	Cat # 5208
Proteome profiler human protease/protease inhibitor array	R&D systems	Cat # ARY025
RIPA lysis buffer	TCI Chemicals	Cat # R0246
HALT protease inhibitor cocktail	ThermoFisher Scientific	Cat # 1861278
EveryBlot Blocking buffer	Biorad	Cat # 12010020
Pierce ECL substrate	ThermoFisher Scientific	Cat # 34577 and 34095
Gelatin	Sigma-Aldrich	Cat #G1890
Coomassie Brilliant Blue R-350	GE Healthcare	Cat # 17-0518-01
Human MMP-12 ELISA	Invitrogen	Cat # EH327RB
Human ADAM-9 DuoSet ELISA	R&D Systems	Cat # DY939
Human TIMP-1 DuoSet ELISA	R&D Systems	Cat #DY970
Human TIMP-2 DuoSet ELISA	R&D Systems	Cat # DY971
Human MMP-9/TIMP-1 DuoSet ELISA	R&D Systems	Cat # DY1449
Human Cystatin C DuoSet ELISA	R&D Systems	Cat # DY1196
Human Cathepsin D DuoSet ELISA	R&D Systems	Cat # DY1014
Human Serpin E1 DuoSet ELISA	R&D Systems	Cat # DY1786
Human TNF-alpha DuoSet ELISA	R&D Systems	Cat # DY210
Human IL-1 beta DuoSet ELISA	R&D Systems	Cat # DY201
Human IL-6 DuoSet ELISA	R&D Systems	Cat # DY206
Human CCL18/PARC DuoSet ELISA	R&D Systems	Cat # DY394
Subcellular Protein Fractionation Kit for Cultured cells	ThermoFisher Scientific	Cat # 78840

**Software and algorithms**		

DIVA software	BD Biosciences	N/A
FlowJo, version10	LLC	N/A
Imaris software	Andor Technology	N/A
ImageJ software version1.53	ImageJ	N/A
ImageQuant TL software	GE Healthcare	N/A
GraphPad Prism software version9.4.0	Prism	N/A

**Other**		

Zeiss Axiovert 200M	Carl Zeiss	N/A
Leica DMi8, Dragonfly 200	Andor	N/A
BD LSR Fortessa X20 Flow cytometer	BD Biosciences	N/A
Vilber Lourmat Fusion system	Labtech International	N/A
Fluorescence spectrophotometer (Clariostar)	GE Healthcare	N/A

### Experimental model and study participant details

#### Primary cell cultures

The use of human primary macrophages was approved by the Red Cross Biobank (RKOV_19006).

### Method details

#### Macrophage differentiation from human monocytes

Human peripheral blood mononuclear cells (PBMCs) were isolated from buffy coats derived from blood donated by healthy volunteers (Blood Transfusion Center of the Belgian Red Cross, Mechelen, Belgium), by density gradient centrifugation with Pancoll human (Cat # P04-601000, Pan Biotech). Human monocytes were purified from PBMCs by adherence for 1.5 h at 37°C, 5% CO_2_. The monolayer was washed to remove non-adherent cells. Monocytes were differentiated into macrophages in complete RPMI 1640 GlutaMAX medium (Cat # 61870036, ThermoFisher Scientific) supplemented with 10% heat-inactivated fetal bovine serum (FBS) (Cat # F7524, Sigma-Aldrich) and 1% PenStrep solution (Cat # 15070063, ThermoFisher Scientific) in the presence of M-CSF/CSF-1 (100 ng/mL, Cat # 300-25, PeproTech) for 4 days at 37°C, 5% CO_2_. Resting macrophages (further referred to as M(M-CSF)) were generated by stimulating the macrophages for 3 additional days with M-CSF (25 ng/mL). Inflammatory macrophage differentiation (further referred to as M(LPS/IFN-γ)) was induced by stimulating the macrophages for 3 additional days with LPS from *Escherichia coli* O111:B4 (50 ng/mL, Cat # L4391, Sigma-Aldrich) and IFN-γ (25 ng/mL, Cat # 300-02, PeproTech). Immunosuppressive differentiation (further referred to as M(IL-4)) was obtained by stimulating the macrophages for 3 additional days with IL-4 (25 ng/mL, Cat # 200-04, PeproTech). Twenty-four hours before macrophage collection and analysis, FBS in the differentiation medium was replaced with 20% KnockOut Serum Replacement (Cat # 10828028, ThermoFisher Scientific) to avoid the influence of proteases and protease inhibitors in the subsequent analysis. Supernatants were collected and cells were washed with ice-cold PBS and scraped off the surface. Cell scraping was chosen over enzymatic harvesting techniques to avoid any influence of the proteases used for enzymatic harvesting on our analysis.

#### Flow cytometry

M(M-CSF), M(LPS/IFN-γ), and M(IL-4) were incubated with human fragment crystallizable receptor (FcR) blocking reagent (Cat # 130059901, Miltenyi Biotec) and extracellularly stained with anti-CD80 BV510 (1:40, clone 2D10, Cat # 305233, Biolegend), anti-CD86 PE-Cy7 (1:40, clone BU63, Cat # 374209, Biolegend), anti-CD206 BC786 (1:125, clone 19.2, Cat # 740999, BD Bioscience), anti-CD209 BV421 (1:66, clone 9E98A, Cat # 330117, Biolegend), anti-HLA-DR APC (1:100, clone L243, Cat # 307622, Biolegend), anti-CD163 PE (1:400, clone GHI/61, Cat # 333606, Biolegend), anti-LRP-1 PE (1:100, clone A2MR-alpha-2, Cat # 12-0919-42, Invitrogen), anti-LRP-2 Alexa Fluor 647 (1:33, clone 545606, Cat # FAB9578R, R&D Systems), anti-EMMPRIN Alexa Fluor 488 (1:800, clone HIM8, Cat # 306207, Biolegend), anti-CD44 APC/Fire 450 (1:40, clone BJ18, Cat # 338818, Biolegend), anti-APP1 Alexa Fluor 488 (1:100, clone 22C11, Cat # MAB348A4, Merck), anti-CD63 APC (1:25, clone H5C6, Cat # 353008, Biolegend), anti-CD74 Superbright 436 (1:40, clone 5–329, Cat # 62-0748-42, Invitrogen), anti-Ku70/80 PE (1:33, clone KU729, Cat # 2547-MSM1-PE-100T, Neobiotechnologies). Dead cells were excluded using Fixable Viability Stain 620 (1:50,000, Cat # 564996, BD Biosciences) or Zombie Aqua (1:20,000, Cat # 423102, Biolegend). Flow cytometry was performed on the BD LSR Fortessa X20 equipped with DIVA software. Results were analyzed with FlowJo (LLC, V10). Gating strategy can be found in Figure S1.

#### Phagocytosis by flow cytometry

M(M-CSF), M(LPS/IFN-γ) and M(IL-4) were incubated with pHrodo Red *S. aureus* bioparticles (Cat # A10010, Invitrogen) according the manufacturer’s instructions. PE positive macrophages represent the macrophage population that phagocytosed pHrodo Red *S. aureus* bioparticles. The phagocytosis index was measured as the percentage PE positive macrophages among all macrophages.

#### Light microscopy and immunofluorescence

##### Cell shape and morphology

The morphology of M(M-CSF), M(LPS/IFN-γ) and M(IL-4) was visualized by using a light microscope (Zeiss Axiovert 200M, Carl Zeiss). For immunofluorescent staining, cells were fixed in Hank’s balanced salt solution (HBSS Ca^2+^/Mg 10×, Cat # 14065-049, ThermoFisher Scientific), 0.35 g/L NaHCO_3_ (Cat # 13433, Honeywell Riedel-de-Haën) and 1 g/L BSA (Cat # T8444.4 protease free, Carl Roth) in Milli-Q (MQ) supplemented with 4% paraformaldehyde (32% PFA, Cat # 047377.9M, Thermo Fisher Scientific) for 1 h at RT. Next, cells were washed with HBSS buffer, followed by permeabilization with 0.1% Triton X-100 for 1 h at RT. Cells were rinsed and then immersed for 2 h in blocking buffer (10% FBS and 5% FcR Blocking agent) at RT. CellMask Green Actin Tracking Stain (Cat # A57243, Invitrogen) and Hoechst 33342 (Cat # 62249, Invitrogen) were used to stain the cytoskeleton and nuclei, respectively. Stained macrophages were covered with a few drops of ProLong diamond mounting medium (Cat # P36961, ThermoFisher Scientific) prior to microscopy analysis (Leica DMi8, Dragonfly 200, Andor). Images were analyzed with the Imaris software.

##### Subcellular tracking of MMP-9, TIMP-1 and the MMP-9/TIMP-1 complex

Recombinant human proMMP-9 was produced using a baculoviral expression system in Sf9 cells and purified from the expression medium by gelatin affinity chromatography (previously described^38^ by our research group). MMP-9, BSA and TIMP-1 were fluorescently labeled with Alexa Fluor (AF)546 (Alexa Fluor 546 Antibody Labeling Kit, Cat # A20183, ThermoFisher Scientific) and/or AF647 (Alexa Fluor 647 Antibody Labeling Kit, Cat # A20186, ThermoFisher Scientific) to track their subcellular presence over time by using fluorescence microscopy. Recombinant TIMP-1 and the TIMP-1 variants were produced as described by Schoeps et al*.*^65^ Briefly, TIMP-1 was expressed in HEK293F cells and supernatants was collected for purification. TIMP-1 was purified using a three-step purification protocol consisting of AIEX, CIEX, and SEC chromatography columns. TIMP-1 containing fractions of the SEC column were pooled. Purity was analyzed using silver stained and proper glycosylation was analyzed using PNGaseF digest. 10 nM of AF546-labeled MMP-9, 10 nM of AF546-labeled BSA and/or 10 nM of AF647-labeled TIMP-1 were added to the culture medium (RPMI 1640 without phenol red + 20% RS) and incubated for 0 min, 30 min, 1 h, 2 h, 6 h, 8 h and 24 h. 30 min prior to imaging, cell nuclei and F-actin were stained with 10 μg/mL Hoechst 33342 and CellMask Green Actin Tracking Stain. To test if uptake was LRP-1-mediated, 100 nM RAP (Cat # 4296-LR, R&D Systems) was added 30 min prior to the addition of AF546-labeled MMP-9. Stained cells were imaged by confocal microscopy (Leica DMi8, Dragonfly 200, Andor) and analyzed with the Imaris software and the ImageJ software 1.53.

#### Quantitative RT-PCR

For qRT-PCR analysis, total RNA from macrophages was isolated using RNeasy mini kit (Cat # NC9677589, Qiagen) according to the manufacturer’s instructions. Reverse transcription for subsequent analysis of mRNA expression levels was performed using the MultiScribe Reverse Transcriptase (Cat # 4311235, Invitrogen) and random primers (Cat # 4319979, Invitrogen). qRT–PCR was performed using a TaqMan gene expression assay (Applied Biosystems) on a 7500 Real-Time PCR System Apparatus. The relative mRNA expression was calculated using the 2-ΔΔCt method and normalized to GAPDH expression of M(M-CSF). Primers used for qRT-PCR are summarized in Table S1.

#### Substrate-based protease activity assays

The activity of proteases present in supernatants and cell lysates of M(M-CSF), M(LPS/IFN-γ) and M(IL-4) was measured by substrate-based protease activity assays. The assay was performed in a total volume of 150 μL for supernatants and 100 μL for cell lysates in wells of 96-well black plates using the fluorescent peptide substrates Mca-PLGL-Dpa-AR-NH2 (10 μM, Cat # ES001, R&D Systems), Mca-PLAQAV-Dpa-RSSSR-NH2 (10 μM, Cat # ES003, R&D systems) and Z-LR-AMC (10 μM, Cat # ES008, R&D systems). Appropriate amounts of Mca-PLGL-Dpa-AR-NH2 and Mca-PLAQAV-Dpa-RSSSR-NH2 were resuspended in MMP assay buffer (50 mM Tris, 150 mM NaCl, 5 mM CaCl_2_, 0.01% Tween 20, pH 7.5), whereas Z-LR-AMC was resuspended in cathepsin assay buffer (50 mM NaCl, 50 mM KH_2_PO_4_, 2 mM EDTA, 2 mM DDT, pH 5.5). Where indicated, EDTA (100 mM, Cat # 20302.260, VWR), pepstatin A (0.7 μg/mL, Cat # 1190, R&D systems) or E 64 (10 μg/mL, Cat # 5208, R&D systems) were added. Initial reaction velocity was monitored continuously at λex = 320 nm and λem = 400 nm for Mca-PLGL-Dpa-AR-NH2 and Mca-PLAQAV-Dpa-RSSSR-NH2, and at λex = 380 nm and λem = 460 nm for Z-LR-AMC using a fluorescence spectrophotometer (Clariostar) at 37°C.

#### Proteome profiler array

Proteome profiler human protease/protease inhibitor arrays were purchased from R&D systems (Cat # ARY025). 300 μL of supernatants and 110 μg proteins of total cell lysate (in RIPA lysis buffer (Cat # R0246, TCI Chemicals) containing 1% of HALT protease inhibitor cocktail (Cat # 1861278, ThermoFisher Scientific)) were loaded on the arrays according to the manufacturer’s protocols. Each membrane was developed using the Vilber Lourmat Fusion system (Labtech International). For analysis, the intensity of each dot was determined using ImageJ software, and the means of two dots corresponding to one defined protease/protease regulators were normalized to the means of all six reference spots. Relative expression levels of proteases and protease regulators were compared between M(LPS/IFN-γ) and M(IL-4). If the difference in expression was more than 20%, these analytes were selected for further analysis (Figures S2 and S3).

#### SDS PAGE, Western blot and gelatin *in gel* zymography

For Western blot analyses, equal amounts of protein were resuspended in reducing loading buffer (125 mM Tris-HCl pH 6.8, 10% β-mercaptoethanol, 4% SDS, 20% glycerol, 0.1% bromophenol blue) and incubated for 10 min at 80°C. Samples were separated in 4–20% or 10–20% Tris-Glycine gels (precast Novex Tris-Glycine gels, ThermoFisher Scientific). Proteins in gels were transferred onto PVDF membranes using the Trans-Blot Turbo Transfer system with associated materials and protocols (Cat # 1704150, Biorad). After protein transfer, membranes were blocked for 5 min in EveryBlot Blocking buffer (Cat # 12010020, Biorad) and incubated with anti-MMP-9 (Cat # AB911, R&D systems), anti-MMP-12 (Cat # AF917, R&D systems), anti-ADAM-9 (Cat # DY939, Detection antibody of Human DuoSet ELISA, R&D systems), anti-SP1 (Cat # 5931, Cell Signaling), anti-β-actin (Cat # 20536-1, Proteintech), anti-H3 (Cat # 9715, Cell Signaling), anti-CD44 (Cat # 3570, Cell Signaling), anti-HSP90 (Cat # MAB3286, R&D systems), anti-TIMP-1 (Cat # 8946S, Cell Signaling) or anti-A2M (Cat # AF1938, R&D Systems) antibodies diluted in 10% EveryBlot Blocking buffer in TBS-T (1× TBS containing 0.1% Tween 20) overnight at 4°C. Membranes were washed three times with TBS-T at RT and incubated for 1 h with secondary anti-goat IgG HRP-conjugated antibody solution (Cat # PI-9500, Vector Labs), anti-rabbit IgG HRP-conjugated antibody solution (Cat # 711-035-152, Jackson ImmunoResearch) or anti-mouse IgG HRP-conjugated antibody solution (Cat # 115-035-071, Jackson ImmunoResearch) at RT. Membranes were washed three times with TBS-T and bands were visualized using the Pierce ECL substrate (ThermoFisher Scientific). Western blots were imaged using the Vilber Lourmat Fusion system (Labtech International).

To detect protein levels of the gelatinases (pro)MMP-2, and (pro)MMP-9, we used gelatin *in gel* zymography.^66^ Gels were prepared consisting of a 7.5% acrylamide separating gel with 1 mg/mL gelatin (Cat # G1890, Sigma-Aldrich), topped with a 5% stacking gel. Gels were placed in an electrophoresis system with running buffer (25 mM Tris, 192 mM glycine, 0.1% SDS) and samples, prepared in non-reducing loading dye (125 mM Tris-HCl pH 6.8, 4% SDS, 20% glycerol, 0.1% bromophenol blue), were added. After electrophoretic protein separation, gels were washed twice for 20 min in reactivation solution (2.5% Triton X-100). Next, gels were incubated overnight in 10 mM CaCl_2_ and 50 mM Tris–HCl, pH 7.5 at 37°C. Staining was performed with 0.1% Coomassie Brilliant Blue R-350 (Cat # 17-0518-01, GE Healthcare), and zymograms were quantified using ImageQuant TL software (GE Healthcare). Molecular weight was determined by a molecular marker consisting of trimeric proMMP-9, monomeric proMMP-9, and a low-molecular weight proMMP-9 domain deletion mutant lacking the O-glycosylated and hemopexin domains (proMMP-9 ΔOGΔHem).

#### *In situ* gelatin zymography

For *in situ* gelatin zymography, glass slides were coated with 50 μg/mL AF488-labeled gelatin as described above. M(M-CSF), M(LPS/IFN-γ), and M(IL-4) were seeded on gelatin-coated glass slides and incubated for 6 h at 37°C/5% CO_2_. 30 min prior to imaging, cell nuclei and F-actin were stained with 10 μg/mL Hoechst 33342 and CellMask Deep Red Actin Tracking Stain (Cat # A57245, Invitrogen). Stained cells were imaged by confocal microscopy (Leica DMi8, Dragonfly 200, Andor) and analyzed with Imaris software and the ImageJ software 1.53.

#### Sandwich ELISA

MMP-12 (Human MMP-12 ELISA, Cat # EH327RB, Invitrogen), ADAM-9 (Human ADAM-9 DuoSet ELISA, Cat # DY939, R&D Systems), TIMP-1 (Human TIMP-1 DuoSet ELISA, Cat # DY970, R&D Systems), TIMP-2 (Human TIMP-2 DuoSet ELISA, Cat # DY971, R&D Systems), MMP-9/TIMP-1 complex (Human MMP-9/TIMP-1 DuoSet ELISA, Cat # DY1449, R&D Systems), Cystatin C (Human Cystatin C DuoSet ELISA, Cat # DY1196, R&D Systems), Cathepsin D (Human Cathepsin D DuoSet ELISA, Cat # DY1014, R&D Systems), Serpin E1 (Human Serpin E1 DuoSet ELISA, Cat # DY1786, R&D Systems), Tumor Necrosis Factor alpha (TNF-α) (Human TNF-alpha DuoSet ELISA, Cat # DY210, R&D Systems), IL-1β (Human IL-1 beta DuoSet ELISA, Cat # DY201, R&D Systems), IL-6 (Human IL-6 DuoSet ELISA, Cat # DY206, R&D Systems) and CCL-18 (Human CCL18/PARC DuoSet ELISA, Cat # DY394, R&D Systems) concentrations were measured by sandwich ELISA according to the manufacturer’s protocols.

#### Subcellular protein fractionation of macrophages

M(M-CSF), M(LPS/IFN-γ) and M(IL-4) were detached by cell scraping. Cells were washed, and cell pellets with a packed cell volume of 10 μL (equivalent to 1 million cells) were obtained. M(M-CSF), M(LPS/IFN-γ) and M(IL-4) were fractionized into membrane, cytoplasm, cytoskeleton, soluble nuclear and chromatin-bound fractions according to the manufacturer’s protocols using the Subcellular Protein Fractionation Kit for Cultured cells (Cat # 78840, ThermoFisher Scientific). Presence of MPs (MMP-9, MMP-12 and ADAM-9) and inhibitors (TIMP-1, TIMP-2 and A2M) was evaluated by Western blot analysis and sandwich ELISA.

### Quantification and statistical analysis

Data were analyzed using GraphPad Prism software, version 9.4.0 (GraphPad software). Data are presented as mean ± SEM. The number of biological replicates (donors) used is indicated in the corresponding figure legend. Normally distributed data were tested by Shapiro-Wilk test. Groups were compared using a paired t test, one-way ANOVA with Geisser-Greenhouse correction followed by a Holm-Šídák multiple comparison test or two-way ANOVA in the case of normal distribution. In the absence of normal distribution, data were log-transformed and again tested for normal distribution (Shapiro-Wilk test). Groups were compared using a paired t test or one-way ANOVA with Geisser-Greenhouse correction followed by a Holm-Šídák multiple comparison test on log-transformed data. In the absence of normal distribution, a Friedman test followed by Dunn’s multiple comparison test was performed. A *p* value of <0.05 was considered significant throughout. The statistical test used for each data graph can be found in Data S1.

## Supplemental information

Document S1. Figures S1–S7 and Table S1

Data S1. Statistical analysis, related to STAR Methods
