# Supplementary material for: Human monocyte-derived macrophages shift subcellular metalloprotease activity depending on their activation state
Source: iScience. 2024 Oct 16;27(11):111171. doi: 10.1016/j.isci.2024.111171 (PMC11576389; doi:10.1016/j.isci.2024.111171)
Supplement: Document S1. Figures S1–S7 and Table S1 [file mmc1.pdf]

iScience, Volume *27*

Supplemental information

Human monocyte-derived macrophages shift subcellular metalloprotease activity depending on their activation state

Eline Bernaerts, Kourosh Ahmadzadeh, Amber De Visscher, Bert Malengier-Devlies, Daniel Häuβler, Tania Mitera, Erik Martens, Achim Krüger, Lien De Somer, Patrick Matthys, and Jennifer Vandooren

Supplementary Figures

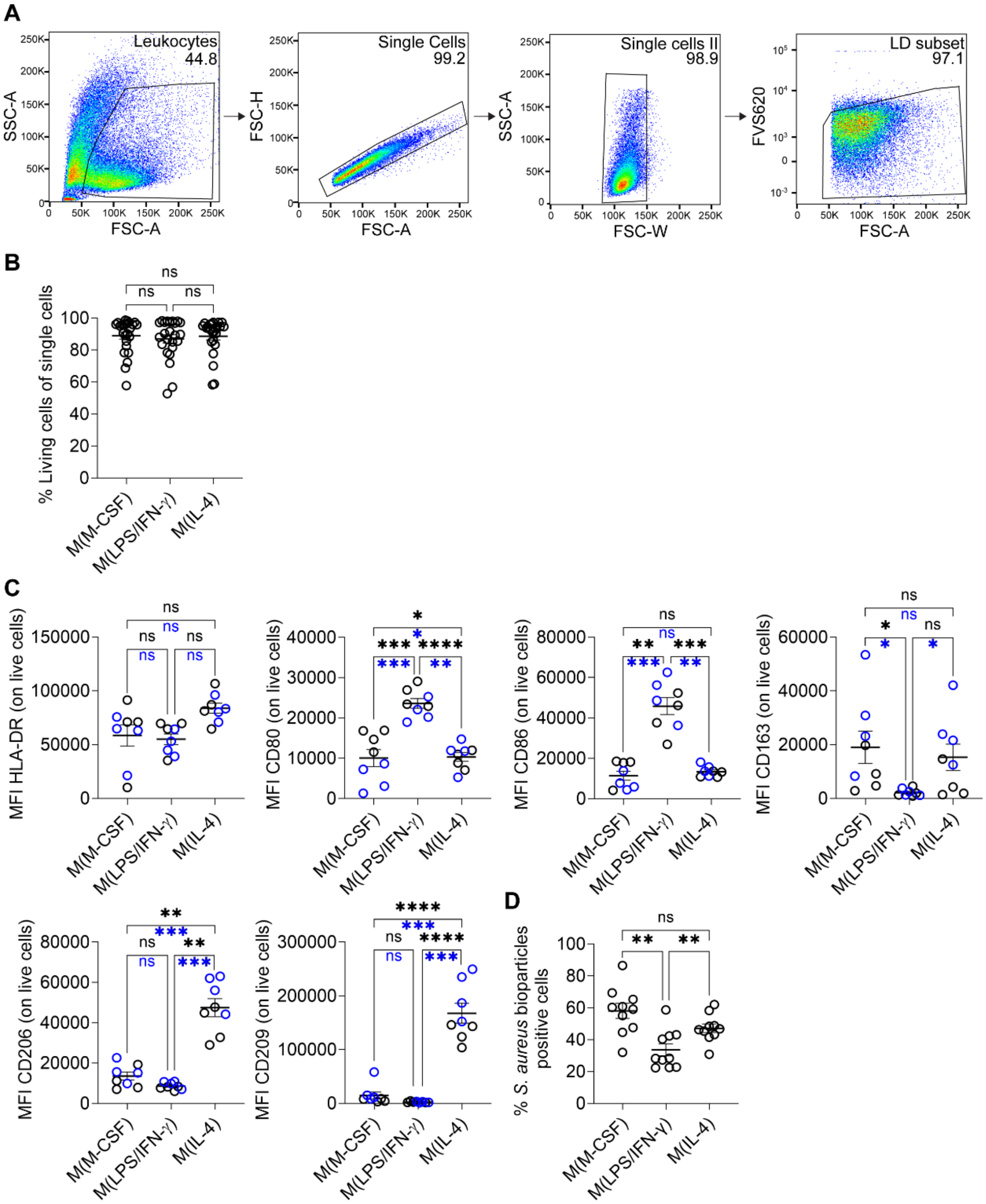

Figure S1. ***In vitro*** **stimulated macrophages, Related to Figure 1 and 2** Monocytes of healthy controls were stimulated with either M-CSF, LPS and IFN-γ or IL-4 into distinct macrophage phenotypes. (**A**) Gating strategy to determine living macrophages (example of M(M-CSF)) by flow cytometry. (**B**) Quantification of percentage living macrophages (of single cells) of different macrophage types. Each data point represents cells from a different donor (n=24). Error bars are mean ± SEM. P values less than 0.05 were considered significant (ns non-significant). (**C**) Flow cytometry analysis of HLA-DR, CD80, CD86, CD163, CD206 and CD209 on M(M-CSF), M(LPS/IFN-γ) and M(IL-4) when cultured with replacement serum (black hollow circles) or FBS (blue hollow circles). Each data point represents cells from a different

donor (n=13). Error bars are mean ± SEM. P-values less than 0.05 were considered significant (* $p < 0.05$; ** $p < 0.01$; *** $p < 0.001$; **** $p < 0.0001$; ns non-significant). (**D**) Quantification of percentage *S. aureus* bioparticles positive macrophages among all macrophages. Each data point represents cells from a different donor (n=10). Error bars are mean ± SEM. P values less than 0.05 were considered significant (**$p < 0.01$; ns non-significant).

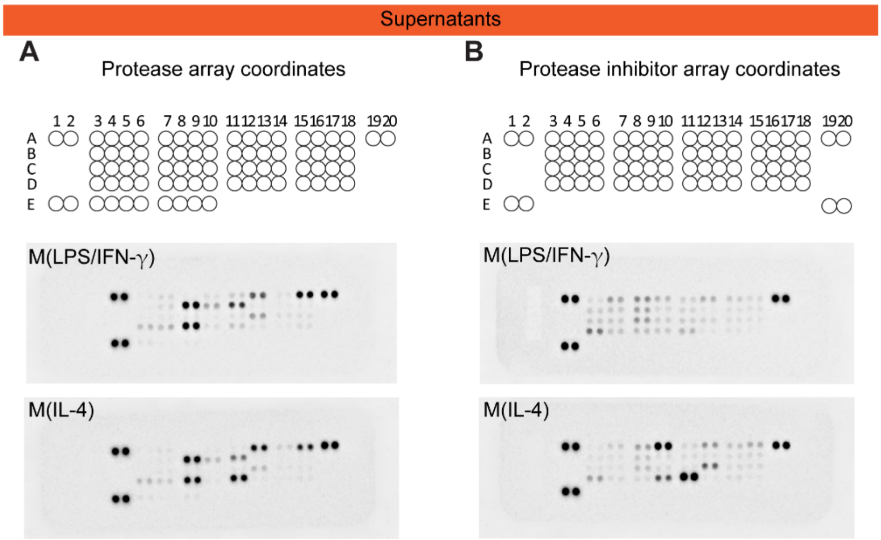

Reference spot	A1, A2, A19, A20, E1, E2
Negative spot	E9, E10
ADAM8	A3, A4
ADAM9	A5, A6
ADAMST1	A7, A8
ADAMST13	A9, A10
Cathepsin A	A11, A12
Cathepsin B	A13, A14
Cathepsin C	A15, A16
Cathepsin D	A17, A18
Cathepsin E	B3, B4
Cathepsin L	B5,B6
Cathepsin S	B7, B8
Cathepsin V	B9, B10
Cathepsin X/Z/P	B11, B12
DPPIV	B13, B14
Kallikrein 3	B15, B16
Kallikrein 5	B17, B18
Kallikrein 6	C3, C4
Kallikrein 7	C5, C6
Kallikrein 10	C7, C8
Kallikrein 11	C9, C10
Kallikrein 13	C11, C12
MMP-1	C13, C14
MMP-2	C15, C16
MMP-3	C17, C18
MMP-7	D3, D4
MMP-8	D5, D6
MMP-9	D7, D8
MMP-10	D9, D10
MMP-12	D11, D12
MMP-13	D13, D14
Neprilysin	D15, D16
Presenilin	D17, D18
Proprotein convertase 9	E3, E4
Proteinase 3	E5, E6
Urokinase	E7, E8

Reference spot	A1, A2, A19, A20, E1, E2
Negative spot	E19, E20
APP	A3, A4
Cystatin A	A5, A6
Cystatin B	A7, A8
Cystatin C	A9, A10
Cystatin E	A11, A12
EMMPRIN	A13, A14
Fetuin B	A15, A16
HAI-1	A17, A18
HAI-2	B3, B4
HE4	B5, B6
Latexin	B7, B8
Lipocalin-1	B9, B10
Lipocalin-2	B11, B12
RECK	B13, B14
Serpin A5	B15, B16
Serpin A8	B17, B18
Serpin A9	C3, C4
Serpin A12	C5, C6
Serpin B5	C7, C8
Serpin B6	C9, 10
Serpin B8	C11, C12
Serpin E1	C13, C14
Serpin F1	C15, C16
Testican 1	C17, C18
Testican 2	D3, D4
TFPI	D5, D6
TFPI-2	D7, D8
TIMP-1	D9, D10
TIMP-2	D11, D12
TIMP-3	D13, D14
TIMP-4	D15, D16
Trappin-2	D17, D18

Figure S2. Levels of secreted proteases and protease regulators of M(LPS/IFN-γ) and M(IL-4), Related to Figure 4 Monocytes of healthy controls were stimulated with either LPS and IFN-γ or IL-4 into distinct macrophage phenotypes. 300 µL of supernatants of M(LPS/IFN-γ) and M(IL-4) were used for each array ((**A**) protease array and (**B**) protease inhibitor array). Expression levels were corrected against the reference spots for each array. Secreted proteases or protease regulators that showed a differential expression of 20% or more between M(LPS/IFN-γ) and M(IL-4) are indicated in purple.

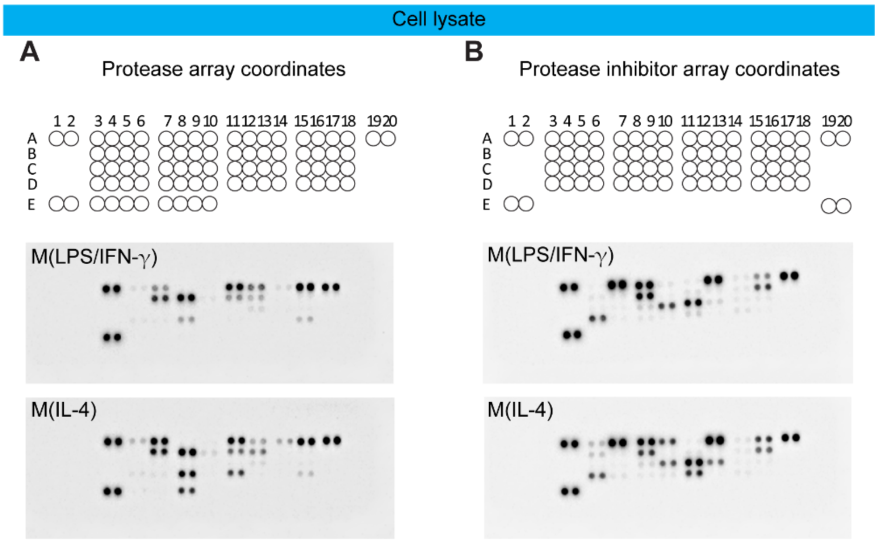

Reference spot	A1, A2, A19, A20, E1, E2
Negative spot	E9, E10
ADAM8	A3, A4
ADAM9	A5, A6
ADAMST1	A7, A8
ADAMST13	A9, A10
Cathepsin A	A11, A12
Cathepsin B	A13, A14
Cathepsin C	A15, A16
Cathepsin D	A17, A18
Cathepsin E	B3, B4
Cathepsin L	B5,B6
Cathepsin S	B7, B8
Cathepsin V	B9, B10
Cathepsin X/Z/P	B11, B12
DPPIV	B13, B14
Kallikrein 3	B15, B16
Kallikrein 5	B17, B18
Kallikrein 6	C3, C4
Kallikrein 7	C5, C6
Kallikrein 10	C7, C8
Kallikrein 11	C9, C10
Kallikrein 13	C11, C12
MMP-1	C13, C14
MMP-2	C15, C16
MMP-3	C17, C18
MMP-7	D3, D4
MMP-8	D5, D6
MMP-9	D7, D8
MMP-10	D9, D10
MMP-12	D11, D12
MMP-13	D13, D14
Neprilysin	D15, D16
Presenilin	D17, D18
Proprotein convertase 9	E3, E4
Proteinase 3	E5, E6
Urokinase	E7, E8

Reference spot	A1, A2, A19, A20, E1, E2
Negative spot	E19, E20
APP	A3, A4
Cystatin A	A5, A6
Cystatin B	A7, A8
Cystatin C	A9, A10
Cystatin E	A11, A12
EMMPRIN	A13, A14
Fetuin B	A15, A16
HAI-1	A17, A18
HAI-2	B3, B4
HE4	B5, B6
Latexin	B7, B8
Lipocalin-1	B9, B10
Lipocalin-2	B11, B12
RECK	B13, B14
Serpin A5	B15, B16
Serpin A8	B17, B18
Serpin A9	C3, C4
Serpin A12	C5, C6
Serpin B5	C7, C8
Serpin B6	C9, 10
Serpin B8	C11, C12
Serpin E1	C13, C14
Serpin F1	C15, C16
Testican 1	C17, C18
Testican 2	D3, D4
TFPI	D5, D6
TFPI-2	D7, D8
TIMP-1	D9, D10
TIMP-2	D11, D12
TIMP-3	D13, D14
TIMP-4	D15, D16
Trappin-2	D17, D18

Figure S3. Levels of cell-associated proteases and protease regulators of M(LPS/IFN-γ) and M(IL-4), Related to Figure 4 Monocytes of healthy controls were stimulated with either LPS and IFN-γ or IL-4 into distinct macrophage phenotypes. 110 µg of cell lysate of M(LPS/IFN-γ) and M(IL-4) were used for each array ((**A**) protease array and (**B**) protease inhibitor array). Expression levels were corrected against the reference spots for each array. Secreted proteases or protease regulators that showed a differential expression of 20% or more between M(LPS/IFN-γ) and M(IL-4) are indicated in purple.

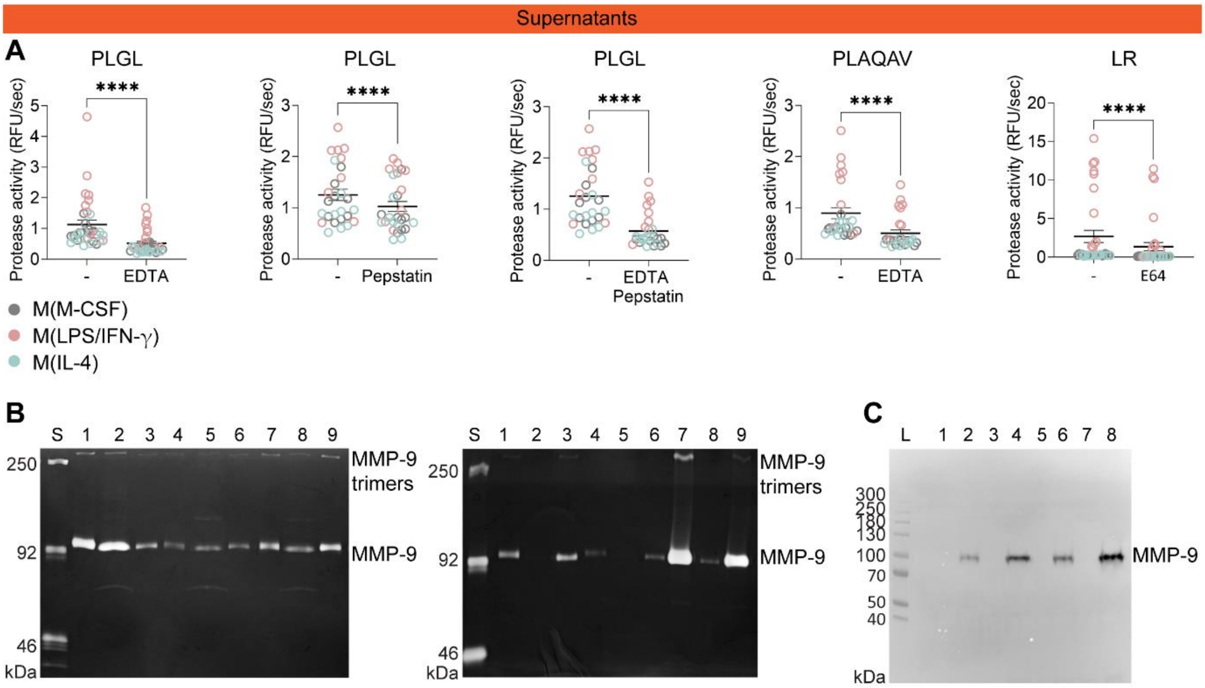

Figure S4. Quantification of secreted proteases of M(M-CSF), M(LPS/IFN-γ) and M(IL-4), Related to Figure 3 and 4 (**A**) Quantification of secreted proteolytic activity of M(M-CSF), M(LPS/IFN-γ) and M(IL-4) by substrate-based degradation assays in the presence of a protease inhibitor. Mca-PLGL-Dpa-AR-NH2 fluorogenic peptide was used to measure proteolytic MMP and Cathepsin D and E activity. EDTA, pepstatin or a combination of both was used to inhibit the proteolytic activity against the Mca-PLGL-Dpa-AR-NH2 fluorogenic peptide. Mca-PLAQAV-Dpa-RSSSR-NH2 fluorogenic peptide was used to measure proteolytic ADAM-9, ADAM-10 and ADAM-17 activity. EDTA was used to inhibit the proteolytic activity against the Mca-PLAQAV-Dpa-RSSSR-NH2 fluorogenic peptide. Z-LR-AMC fluorogenic peptide was used to measure proteolytic Cathepsin B, L and V activity. E64 used to inhibit the proteolytic activity against the Z-LR-AMC fluorogenic peptide. Each data point represents cells from a different donor (at least n=9). Gray circles represent M(M-CSF), red circles represent M(LPS/IFN-γ) and green circles represent M(IL-4). Error bars are mean ± SEM. P values less than 0.05 were considered significant (**** $p < 0.0001$). (**B**) Left: Quantification of MMP-9 present in cell culture supernatants of M(M-CSF) (lane 1, 4, 7, diluted 1/25), M(LPS/IFN-γ) (lane 2, 5, 8, undiluted) and M(IL-4) (lane 3, 6, 9, diluted 1/25) was determined by gelatin *in gel* zymography (left). Right: Equal amounts of cell culture supernatants of M(M-CSF) (lane 1, 4, 7, diluted 1/25), M(LPS/IFN-γ) (lane 2, 5, 8, diluted 1/25) and M(IL-4) (lane 3, 6, 9, diluted 1/25) were loaded onto a gelatin zymogram. Recombinant standard mixture (S) was loaded as internal reference. It indicates multimeric proMMP-9, monomeric proMMP-9 and a low-molecular weight proMMP-9 domain deletion mutant lacking the O-glycosylated and hemopexin domains (proMMP-9 ΔOGΔHem). (**C**) Anti-MMP-9 Western blot of cell culture

supernatants (equal amounts) of M(LPS/IFN-γ) (lane 1, 3, 5 and 7) and M(IL-4) (lane 2, 4, 6 and 8). Spectra HR (L) was loaded as internal reference.

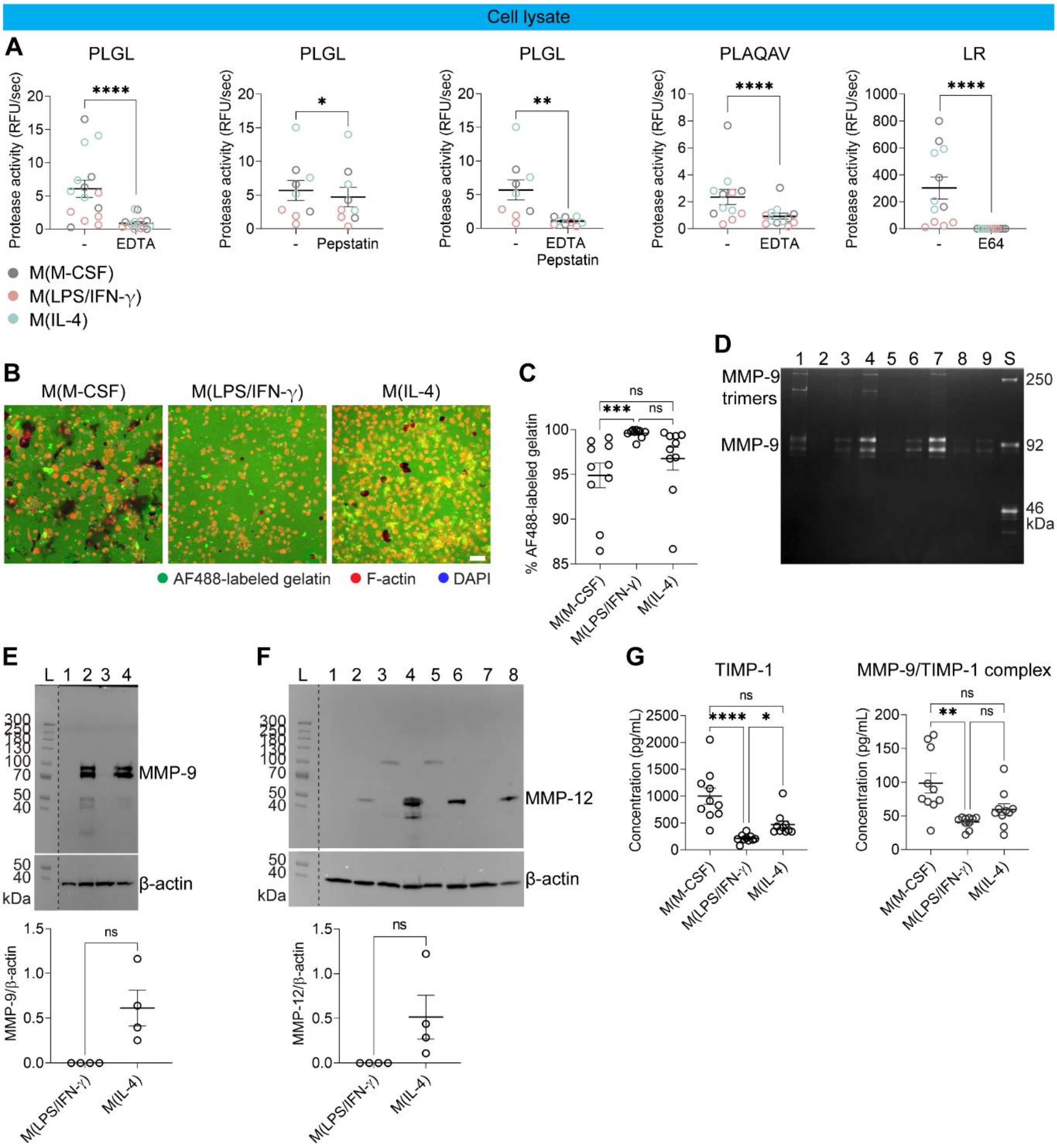

Figure S5. Quantification of cell-associated protease activity and proteases of M(M-CSF), M(LPS/IFN-γ) and M(IL-4), Related to Figure 3 and 4 (**A**) Quantification of cell-associated proteolytic activity of M(M-CSF), M(LPS/IFN-γ) and M(IL-4) by substrate-based degradation assays in presence of a protease inhibitor. Mca-PLGL-Dpa-AR-NH2 fluorogenic peptide was used to measure proteolytic MMP and Cathepsin D and E activity. EDTA, pepstatin or a combination of both was used to inhibit the proteolytic activity against the Mca-PLGL-Dpa-AR-NH2 fluorogenic peptide. Mca-PLAQAV-Dpa-RSSSR-NH2 fluorogenic peptide was used to measure proteolytic ADAM-9, ADAM-10 and ADAM-17 activity. EDTA was used to inhibit the proteolytic activity against the Mca-PLAQAV-Dpa-RSSSR-NH2 fluorogenic peptide. Z-LR-AMC fluorogenic peptide was used to measure proteolytic Cathepsin B, L and

V activity. E64 used to inhibit the proteolytic activity against the Z-LR-AMC fluorogenic peptide. Each data point represents cells from a different donor (at least n=3). Gray circles represent M(M-CSF), red circles represent M(LPS/IFN-γ) and green circles represent M(IL-4). Error bars are mean ± SEM. P values less than 0.05 were considered significant (* $p < 0.05$; ** $p < 0.01$; **** $p < 0.0001$)**.** (**B**) Representative fluorescent images of *in situ* gelatin zymography of M(M-CSF), M(LPS/IFN-γ) and M(IL-4). M(M-CSF), M(LPS/IFN-γ) and M(IL-4) are seeded on AF488-gelatin coated slides. After 6 hours of incubation, gelatinolytic activity on the cell surface of M(M-CSF), M(LPS/IFN-γ) and M(IL-4) is detected by black zones on a fluorescent background (green). Structure of cells is visualized by F-actin staining (Phalloidin, red) and nuclei are visualized by Hoechst 33342 staining (DAPI, blue). Images are representative of 10 different donors. The scale bar represents 50 µm. (**C**) Quantification of gelatinolytic activity on cell surface of M(M-CSF), M(LPS/IFN-γ) and M(IL-4). Each data point represents cells from a different donor (n=10). Error bars are mean ± SEM. P values less than 0.05 were considered significant (*** $p < 0.001$; ns non-significant). (**D**) Gelatin *in gel* zymography of cell lysates of M(M-CSF) (lane 1, 4 and 7), M(LPS/IFN-γ) (lane 2, 5 and 8) and M(IL-4) (lane 3, 6 and 9). Recombinant standard mixture (S) was loaded as internal reference and used for quantification (Figure 4). It indicates multimeric proMMP-9, monomeric proMMP-9 and a low-molecular weight proMMP-9 domain deletion mutant lacking the O-glycosylated and hemopexin domains (proMMP-9 ΔOGΔHem). (**E**) Quantification of MMP-9 present in cell lysates of M(LPS/IFN-γ) (lane 1 and 3) and M(IL-4) (lane 2 and 4) by Western blot analysis. Spectra HR (L) was loaded as internal reference. Data were normalized to β-actin. Each data point represents cells from a different donor (n=4). Error bars are mean ± SEM. P values less than 0.05 were considered significant (ns non-significant)**.** (**F**) Quantification of MMP-12 present in cell lysates of M(LPS/IFN-γ) (lane 1, 3, 5 and 7) and M(IL-4) (lane 2, 4, 6 and 8) by Western blot analysis. Spectra HR (L) was loaded as internal reference. Data were normalized to β-actin. Each data point represents cells from a different donor (n=4). Error bars are mean ± SEM. P values less than 0.05 were considered significant (ns non-significant)**.** (**G**) Quantification of TIMP-1 and MMP-9/TIMP-1 complex present in cell lysate of M(M-CSF), M(LPS/IFN-γ) and M(IL-4) by ELISA. Each data point represents cells from a different donor (n=10). Error bars are mean ± SEM. P values less than 0.05 were considered significant (* $p < 0.05$; ** $p < 0.01$; **** $p < 0.0001$; ns non-significant).

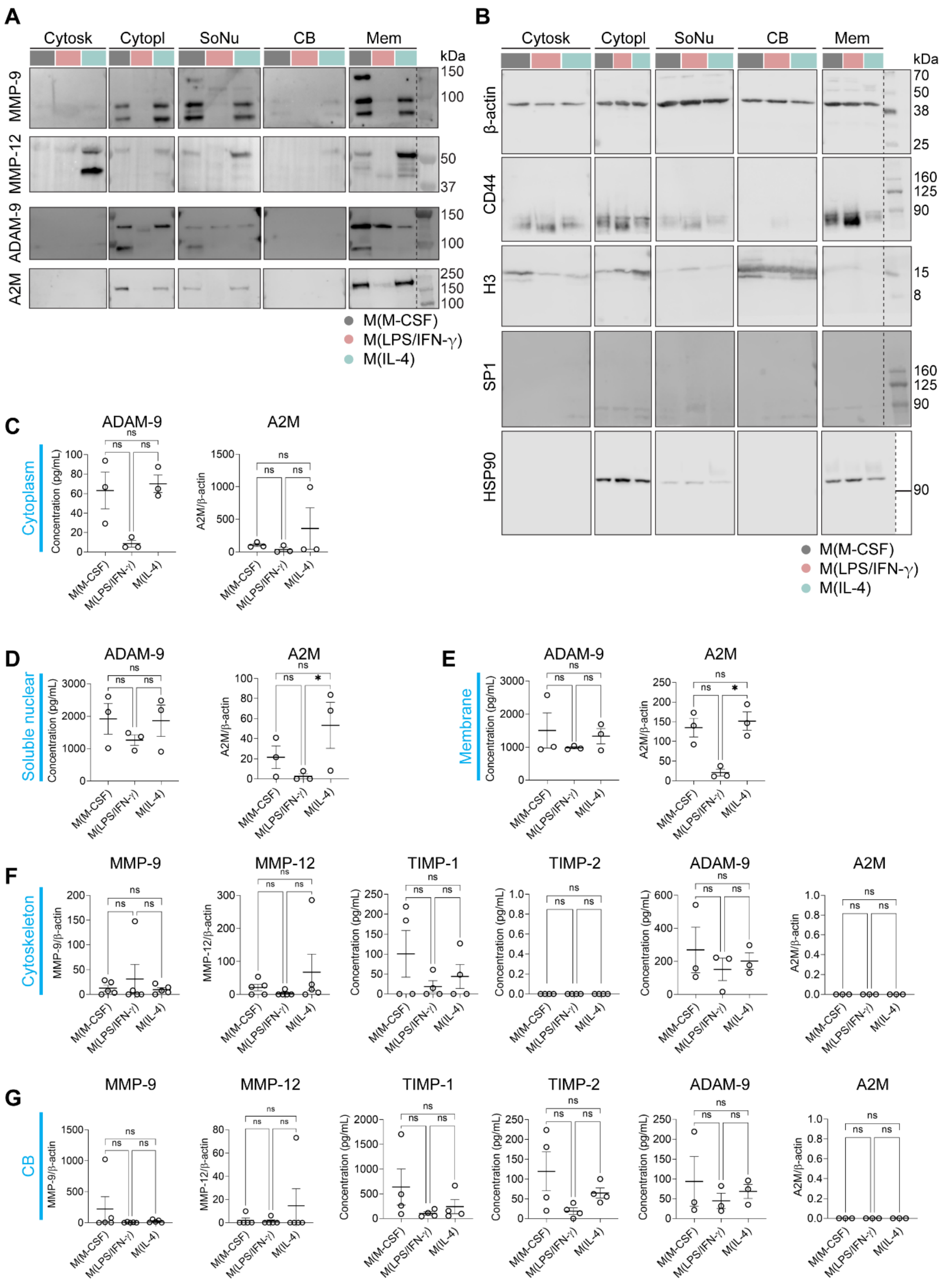

Figure S6. Analysis of proteases and protease inhibitors in macrophage cell compartments, Related to Figure 5 (**A**) Cellular protein fractions of M(M-CSF) M(LPS/IFN-γ) and M(IL-4), including cytoskeleton (Cytosk), cytoplasm (Cytopl), soluble nuclear (SoNu),

chromatin-bound (CB) and membrane (Mem) protein fraction were analyzed by immunoblotting for MMP-9, MMP-12, ADAM-9 and A2M. (**B**) Following fraction controls were included β-actin, CD44, H3, SP1 and HSP90. (**C**) Quantification of ADAM-9 (n=3) and total A2M (n=3) in cytoplasm protein fraction of M(M-CSF), M(LPS/IFN-γ) and M(IL-4), determined by Western blot analysis (data were normalized to β-actin) and ELISA. (**D**) Quantification of ADAM-9 (n=3) and total A2M (n=3) in soluble nuclear protein fraction of M(M-CSF), M(LPS/IFN-γ) and M(IL-4), determined by Western blot analysis (data were normalized to β-actin) and ELISA. (**E**) Quantification of ADAM-9 (n=3) and total A2M (n=3) in membrane protein fraction of M(M-CSF), M(LPS/IFN-γ) and M(IL-4), determined by Western blot analysis (data were normalized to β-actin) and ELISA. (**F**) Quantification of total MMP-9 (n=5), total MMP-12 (n=5), ADAM-9 (n=3), TIMP-1 (n=4), TIMP-2 (n=4), total A2M (n=3) in cytoskeleton protein fraction of M(M-CSF), M(LPS/IFN-γ) and M(IL-4), determined by Western blot analysis (data were normalized to β-actin) and ELISA. (**G**) Quantification of total MMP-9 (n=5), total MMP-12 (n=5), ADAM-9 (n=3), TIMP-1 (n=4), TIMP-2 (n=4), total A2M (n=3) in chromatin-bound protein fraction of M(M-CSF), M(LPS/IFN-γ) and M(IL-4), determined by Western blot analysis (data were normalized to β-actin) and ELISA. Each data point represents cells from a different donor. Error bars are mean ± SEM. P values less than 0.05 were considered significant (* $p < 0.05$; ns non-significant).

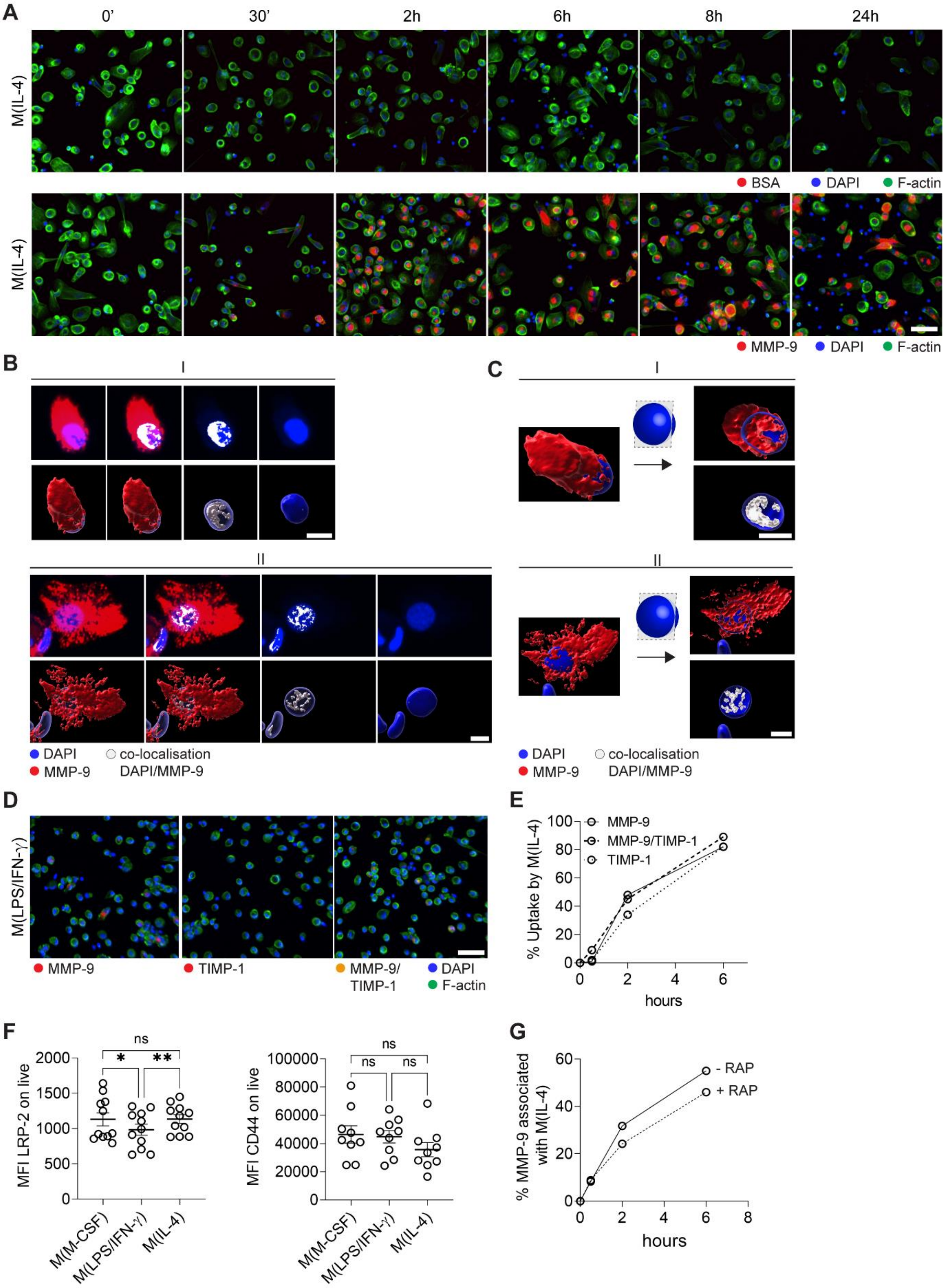

Figure S7. Cellular entry may dictate intracellular MMP-9 localization in M(IL-4), Related to Figure 6 (**A**) Representative immunofluorescent images of M(IL-4) over time (0 minutes to 24 hours) upon incubation with exogenous fluorescently labeled recombinant BSA (red, top

panels) or exogenous fluorescently labeled recombinant human MMP-9 (red, bottom panels). Structure of cells is visualized by F-actin staining (Phalloidin, green) and nuclei are visualized by Hoechst 33342 staining (DAPI, blue). Images are representative of 8 different donors. The scale bar represents 50 µm. (**B**) Confocal images of M(IL-4) (examples I and II) incubated with exogenous fluorescently labeled recombinant human MMP-9. The nucleus is shown in blue (DAPI), MMP-9 is shown in red. Regions of MMP-9/DAPI colocalization are shown in white. The top panel shows confocal images; the bottom panel shows a surface depiction of the confocal images. The scale bar represents 10 µm. (**C**) Confocal images of M(IL-4) (examples I and II) incubated with exogenous fluorescently labeled recombinant human MMP-9. The nucleus is shown in blue (DAPI), MMP-9 is shown in red. Regions of MMP-9/DAPI colocalization are shown in white. The left panel shows a surface depiction of MMP-9. The right panels show a coronal cross section (XY plane) of the surface depiction of MMP-9. The scale bar represents 10 µm. (**D**) Representative immunofluorescent images of M(LPS/IFN-γ) upon 2 hours of incubation with exogenous fluorescently labeled recombinant human MMP-9, (red, left panel), TIMP-1 (red, middle panel) or MMP-9/TIMP-1 complex (orange, right panel). Structure of cells is visualized by F-actin staining (Phalloidin, green) and nuclei are visualized by Hoechst 33342 staining (DAPI, blue). Images are representative of 2 different donors. The scale bar represents 50 µm. (**E**) Quantification of the uptake of exogenous fluorescently labeled recombinant MMP-9, TIMP-1 and MMP-9/TIMP-1 complex by M(IL-4), followed over time (0 minutes to 6 hours) for 1 donor. (**F**) Flow cytometry analysis of LRP-2 and CD44 on M(M-CSF), M(LPS/IFN-γ) and M(IL-4). Each data point represents cells from a different donor (at least n=9). Error bars are mean ± SEM. P-values less than 0.05 were considered significant (* $p < 0.05$; ** $p < 0.01$; ns non-significant). (**G**) Quantification of MMP-9 uptake by M(IL-4) in presence of RAP (LRP-1 inhibitor), followed over time (0 minutes to 6 hours) for 1 donor.

Supplementary Tables

Table S1. Primer sequences used for qRT-PCR, Related to STAR Methods

Gene		Sequence
STAT1	Probe	5’-/56-FAM/AACCTTGCA/ZENGAACAGAGAACACGAGAC/3IABkFQ/-3’
	Primer 1	5’-TAGAGCATGAAATCAAGAGCCT-3’
	Primer 2	5’-GATCACTCTTTGCCACACCA-3’
STAT6	Probe	5’-/56-FAM/CTTCCAGCA/ZEN/CCGTTCTGTGTCCT/3IABkFQ/-3’
	Primer 1	5‘-TCAATGACAACAGCCTCAGTA-3’
	Primer 2	5’-ACCATCAAACCACTGCCAA-3’
